# Supplementary material for: Laplacian Spectra of Persistent Structures in Taiwan, Singapore, and US Stock Markets
Source: Entropy (Basel). 2023 May 25;25(6):846. doi: 10.3390/e25060846 (PMC10297551; doi:10.3390/e25060846)
Supplement: Supplementary file 1 [file entropy-25-00846-s001.zip › entropy-2289932-supplementary.pdf]

Supplementary Information

A. Spectral Sequences of Additional Toy Models

A.1. Three Clusters

Continuing, let us consider the three clusters shown in Supplementary Figure A1(left), where the green and blue clusters are separated by a small distance, while the red cluster is separated from the green and blue clusters by a large distance. In Supplementary Figure A1(right), we see a first persistent band gap (orange) corresponding to the gap between the green and blue clusters. Before the green and blue clusters merge at $\epsilon \approx 1$, the number of zero eigenvalues is 3, because all three clusters are distinct. As this gap closes, the second persistent band gap (yellow) can be seen, and this corresponds to the gap between the red cluster and the green-blue supercluster. Over a wider range of ϵ, the number of zero eigenvalues is 2, because the red cluster remains distinct from the green-blue supercluster.

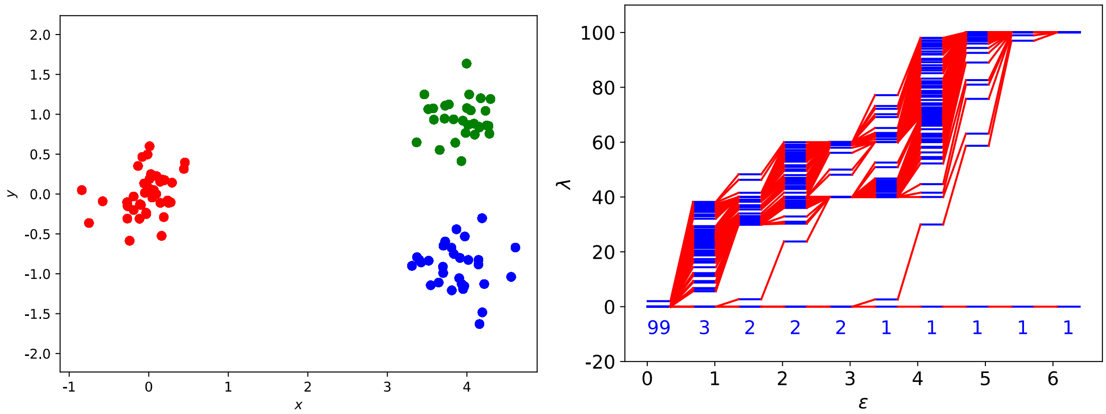

Figure A1: (left) In this example, we have three clusters of points: a red cluster of 40 points, a green cluster of 30 points, and a blue cluster of 30 points. The centers of these clusters are roughly at $(0,0)$, $(3,-1)$, and $(3,1)$ respectively. (right) The spectral sequence of the graph Laplacian L_0 as we increase the filtration parameter ϵ. At each of the sampled values of ϵ, we have 100 eigenvalues (horizontal blue bars). The red lines connect the nth eigenvalue λ_n at ϵ_m with the nth eigenvalue at ϵ_{m+1}. The integer in blue at the bottom of each band of eigenvalues is the number of zero eigenvalues.

At $\epsilon = 1.439$, there are only 2 zero eigenvalues, but the first persistent gap $\Delta\lambda$ is the largest here. The first nonzero eigenvalue is $\lambda_1 = 1.076$, which tells us that the neck between the persistent green and blue clusters consists of no more than 60 links. The second persistent gap $\Delta\lambda$ is largest around $\epsilon = 3$. Thereafter, a narrow neck forms between the persistent red cluster and the green-blue supercluster, as suggested by the small nonzero eigenvalue λ_1 at $\epsilon \approx 3.5$. Thus, when there are persistent clusters at multiple length scales, the spectral signature we expect to see is a succession of persistent gaps.

A.2. Four Clusters with Two Different Gap Sizes

Before we go on to explore other properties of the spectral sequence, let us consider four clusters separated by two different spatial gaps $d_1 < d_2$, i.e., clusters 1 (red) and 2 (green)

are d_1 apart, clusters 3 (blue) and 4 (magenta) are d_1 apart, while the two superclusters are separated d_2. We consider two such configurations with different cluster sizes, to see if a secondary gap will appear away from $\lambda = 0$. As shown in Supplementary Figure A2, in the first case where all four clusters have the same number of points, we see only the first and second persistent gaps. Tracking the first persistent gap as ϵ increases in the first case, we find the four clusters reached the complete network limit at the same time, and have comparable maximum eigenvalues ($\lambda_{\max} \approx 25$). In the second case, when the four clusters have different number of points, we see additional non-persistent gaps at larger eigenvalues λ, over and above the first and second persistent gaps. This happens because the four clusters still reach the complete network limit around the same time, where the band structure is the simplest. However, these clusters have different maximum eigenvalues ($\lambda_{\max} \approx 10, 20, 30, 40$). Surprisingly, the spectral sequence also allows us to tell whether the size distribution of persistent clusters is mono-disperse or polydisperse.

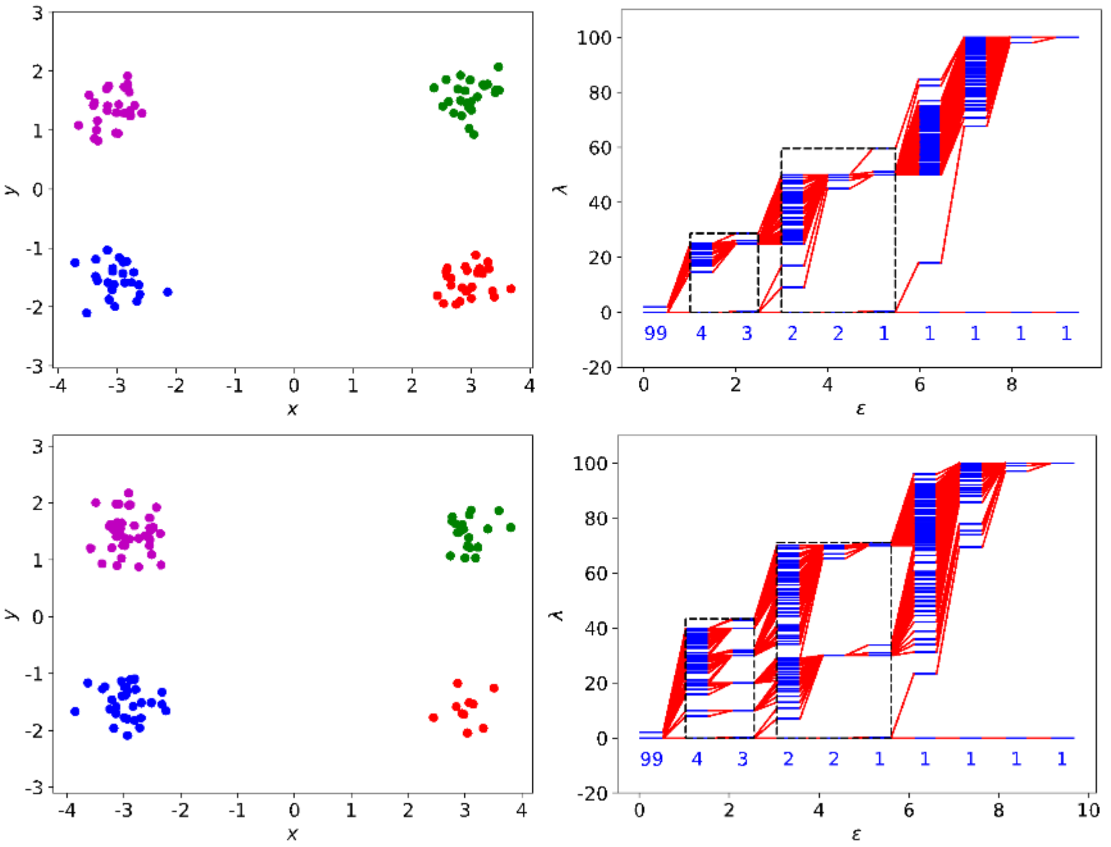

Figure A2: (top left) Four clusters (each with 25 points) at the corners of a rectangle, so that the red and green clusters are close, as are the blue and magenta clusters, but the red-green supercluster is far from the blue-magenta supercluster. (top right) The spectral sequence of this configuration of clusters, showing the first persistent gap (associated with the small separation) and the second persistent gap (associated with the large separation). (bottom left) The same four clusters at the corners of a rectangle, except that the red cluster contains 10 points, the green cluster 20 points, the blue cluster 30 points, and the magenta cluster 40 points. (bottom right) In addition to the first and second persistent gaps (shown as dashed boxes), we find additional gaps due to the different cluster sizes.

As we continue to track the second persistent gap as ϵ increases, we realized that we could

tell that the first case is mono-disperse in terms of superclusters (both 50 points), and the second case involves two superclusters of different sizes (30 points and 70 points). These gaps away from $\lambda = 0$ are non-persistent, because they appear only over very short ranges of ϵ. Non-persistent gaps become apparent only if (a) the clusters or superclusters have different sizes, (b) they have roughly the same density of points, and (c) their size distribution is close to being discrete. If there are many clusters or superclusters, with a broad distribution of sizes, or a broad distribution of densities, then non-persistent gaps may not appear in the spectral sequence.

A.3. Two Clusters at Different Separations

In Section A.1 and Section A.2, we explored different numbers of clusters separated by more than one length scale, but the separations are fixed. Here let us keep the distributions of points in two clusters fixed as shown in Supplementary Figure A3, and vary their separations to understand how the spectral signature changes. As we can see, when this separation is small, the two clusters overlap, and the spectral sequence resembles that from a single cluster. When the two clusters become nearly resolved (only minimal overlap), a weak persistent gap forms. This persistent gap becomes stronger as the separation becomes larger. In the right most spectral sequence of Supplementary Figure A3, we see at $\epsilon \approx 2.5$ that the band of nonzero eigenvalues becomes the narrowest. This is because the two clusters have the same density, and attain the complete network limit simultaneously.

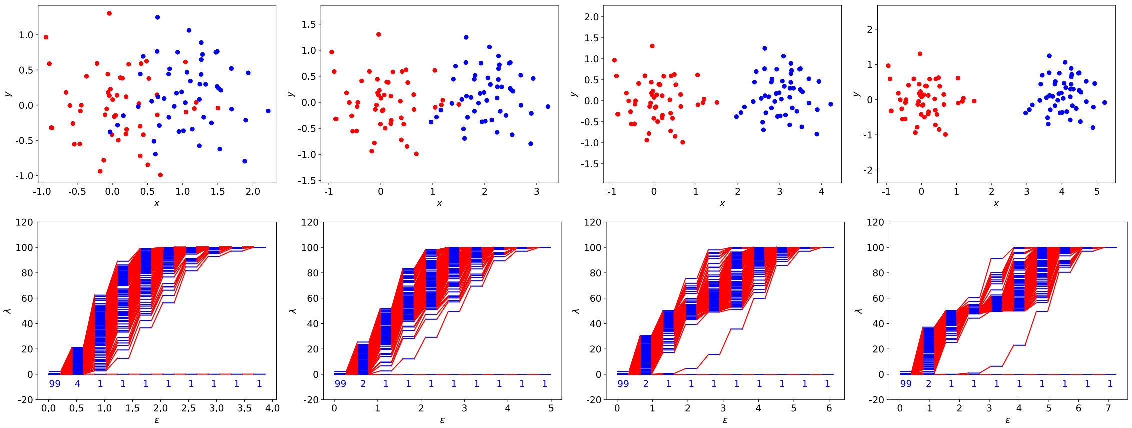

Figure A3: (top row) Two clusters, red and blue, each with fixed distributions of 50 data points. The separation between the two clusters increases going from left to right. (bottom row) The corresponding spectral sequences.

A.4. Two Merging Clusters

Instead of two clusters with rigid distributions of points closing in on each other, we are much more interested in the merging of two clusters through their interactions. When the two clusters interact, their centers approach each other, and their shapes are also likely to become distorted to form a neck. Therefore, in this subsection, we created an artificial dynamics situation of the merging process between two clusters by progressively adding more distortions to the distributions of data points. In the first frame shown in Supplementary Figure A4(a), we have two initially separated clusters, with no neck between them. In the second frame (Supplementary Figure A4(b)), interactions between the two clusters start to

pull some red points toward the blue cluster, and *vice versa*. In other words, the neck is in the process of being formed. Finally, in the third frame (Supplementary Figure A4(c)), the two clusters become connected through the narrow neck formed. In all three frames, the two clusters remain distinguishable.

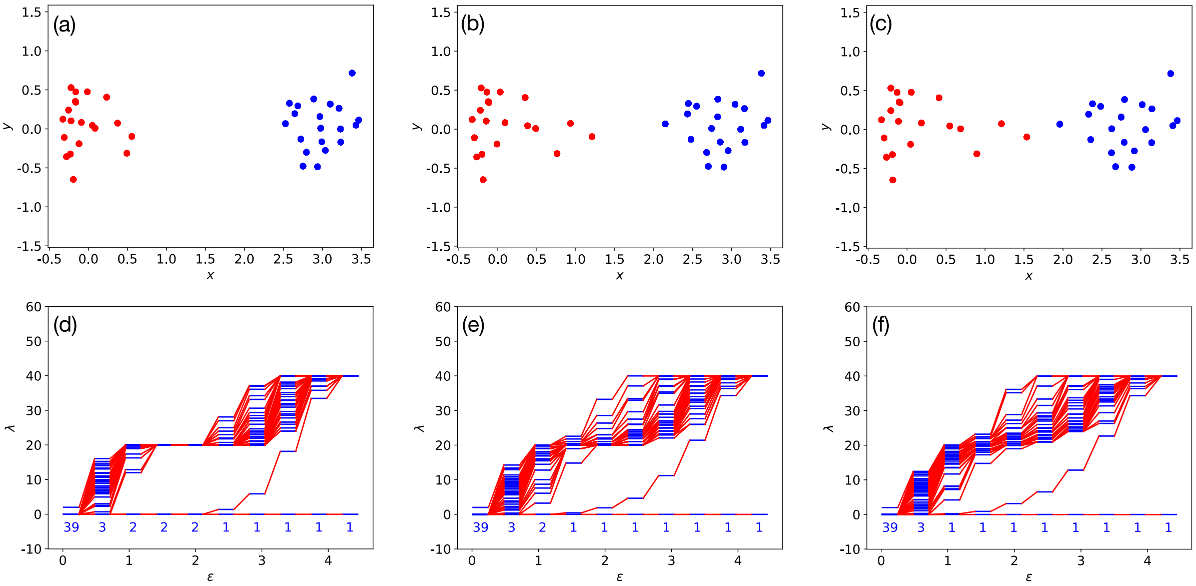

Figure A4: (top row: (a), (b), (c)) An artificial dynamics sequence of two initially separated clusters merging through the formation of a narrow neck between them. (bottom row: (d), (e), (f)) The corresponding spectral sequences.

In Supplementary Figure A4(d), when the two clusters are distinct, the persistent gap disappears only after $\epsilon > 2$. In contrast, before the neck is formed in Supplementary Figure A4(e), the persistent gap started disappearing earlier, at around $\epsilon = 1.5$. Thereafter, the neck forms even earlier in Supplementary Figure A4(f). The most important change is the degeneracy of the zero eigenvalue. In Supplementary Figure A4(d) and Supplementary Figure A4(e), we can always find value(s) of ϵ such that the simplicial complex consisted of two components. In Supplementary Figure A4(f), we found no value of ϵ for which there are two zero eigenvalues. We confirmed this by using more discrete values of ϵ for the filtration. In other words, a two-cluster description of the data points is simply not robust (in the traditional sense, as well as in the spectral sense).

A.5. Two Clusters in a Noisy Background

Finally, let us also consider the situation of two clusters within a noisy background. In Supplementary Figure A5, we show the same two red and blue clusters, with 50 points each, in backgrounds with different noise levels. By noise level, we mean the number of black points (*dust particles*) that are not part of the red or blue clusters. When the noise level is low (5 black points), the persistent gap between the red and blue clusters remains largely unaffected, although the spectral sequence looks like a distribution of data points with two gap scales ($\epsilon \approx 1$ and $\epsilon \approx 2$). However, unlike in Supplementary Figure A1 and Supplementary Figure A2, the first gap scale is not real, in the sense that it is created by the noisy black background points. As the noise level increases, the area of the persistent gap decreases, but the length scale where the two clusters merge remains unchanged. This is reminiscent of doping a semiconductor, where we introduce additional energy levels inside the band gap that remains

more or less the same.

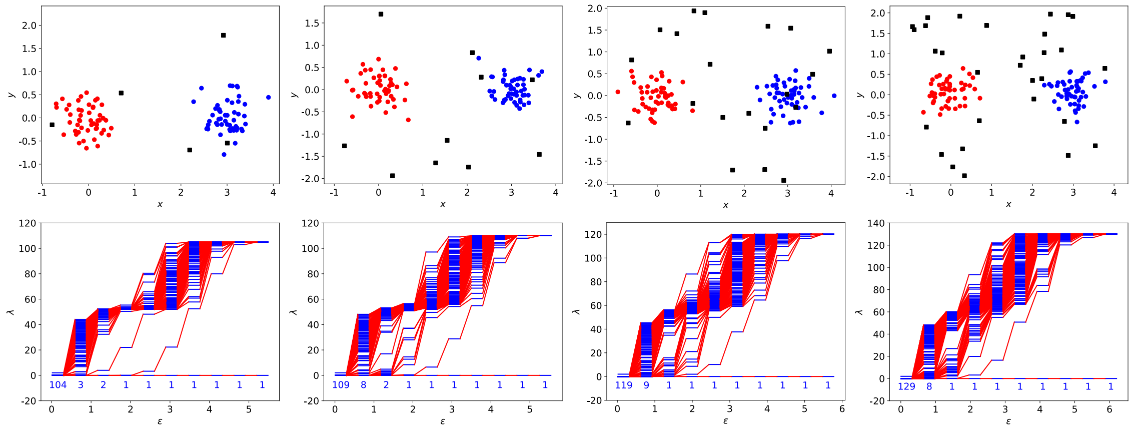

Figure A5: (top row from left to right) Two clusters, with 50 red points and 50 blue points, in noisy backgrounds with 5, 10, 20, and 30 black points. (bottom row) The associated spectral sequences.

B. Fiedler Eigenvector Analysis of Additional Toy Models

B.1. Filtration Sequence for Three Clusters

To ensure that the insights obtained above from the filtration of two clusters is sufficiently general, we also examined the Fiedler vector associated with the filtration of three clusters, as shown in Supplementary Figure A1. At $\epsilon = 1.351$, $\lambda_0 = 0$ is two-fold degenerate. The first eigenvector is associated with cluster 1 (red), while the second eigenvector is the symmetric superposition between cluster 2 (green) and cluster 3 (blue). The Fiedler eigenvector $\vec{u}_1$ associated with $\lambda_1 = 2.685$ is close to the antisymmetric superposition between the green and blue clusters. None of the components in the Fiedler vector are zero, but there are six nodes in $\vec{u}_1$ with smaller weights, with $\{43, 46, 55\}$ belonging to the green cluster, and $\{84, 93, 99\}$ belonging to the blue cluster. However, there appear to be more nodes in the neck. First, nodes in the green cluster with weight $+0.140$ have no links to *any* blue nodes, while those in the blue cluster with weight -0.140 have no links to *any* green nodes. Therefore, the remaining nodes

$$\{40, 43, 44, 46, 47, 48, 49, 53, 54, 55, 58, 59, 62, 63, 64, 65, 68, 69,$$
$$71, 72, 73, 74, 75, 76, 77, 84, 87, 90, 93, 94, 95, 97, 98, 99\}$$

with absolute weights smaller than 0.140 should also be considered neck nodes. However, not all of them are equally important. For example, the green node 43 with weight $+0.045$ is linked to 16 blue nodes, whereas the blue node 98 with weight -0.133 is linked only to 1 green node (43). Therefore, an important takeaway message from this analysis is that the closer the absolute weight of a node to the maximum cluster weight, the closer it is to being a clustered node, and the less it contributes to the neck.

Furthermore, the nodes $\{43, 46, 55, 84, 93, 99\}$ are special: they form a complete bipartite network in which the green nodes $\{43, 46, 55\}$ are linked to all the blue nodes $\{84, 93, 99\}$. We think of these six nodes as the *core of the neck*. If we keep only the green nodes $\{43, 46, 55\}$, the blue nodes can be expanded to include $\{76, 84, 93, 97, 99\}$ and still

produce a complete bipartite network. However, using the expanded list of blue nodes, no more green nodes can be added. On the other hand, if we keep only the blue nodes $\{84, 93, 99\}$, the list of green nodes can be expanded to $\{40, 43, 46, 55, 59, 62, 64\}$ for the bipartite network to be complete. In Figure 14, we show the unexpectedly complex structure of the neck, with $\{43, 46, 55, 84, 93, 99\}$ forming an inner core, while $\{40, 59, 62, 64\}$ and $\{76, 97\}$ form separate outer cores.

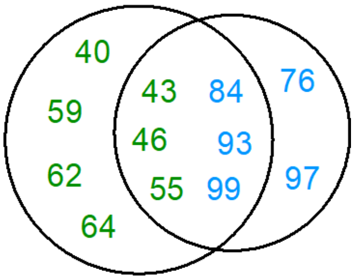

Figure B1: The core structure of the neck between the green cluster 2 and blue cluster 3 for $\epsilon = 1.351$ for the three-cluster data set shown in Supplementary Figure A1. In this figure, we show the core structure in terms of a Venn diagram between a green set of neck nodes and a blue set of neck nodes. The green nodes $\{43, 46, 55\}$ and the blue nodes $\{84, 93, 99\}$ form a complete bipartite network with each other, and together represents the inner core of the neck. The green nodes $\{40, 59, 62, 64\}$ and the blue nodes $\{76, 97\}$ form two outer cores of the neck, in that they form complete bipartite networks only with the inner core.

In line with the distinct neck interpretation proposed in Section 3.4.2, $\{43, 46, 55, 84, 93, 99\}$ belongs completely to the neck n, which is in the process of absorbing more nodes from the green and blue clusters. Therefore, we expect that the complete bipartite core of the neck would enlarge to include $\{40, 59, 62, 64\}$ and $\{76, 97\}$ at a larger value of ϵ. To test this hypothesis, we go to a larger filtration parameter $\epsilon = 2.023$ ($\lambda_1 = 23.772$), where $\lambda_0 = 0$ remains two-fold degenerate, and their eigenvectors continue to represent the distinct red cluster, and the merged green-blue supercluster. At this filtration parameter, the inner core of the neck comprises the green nodes

$$n_G = \{40, 43, 44, 46, 47, 48, 49, 51, 54, 55, 58, 59, 62, 63, 64, 65, 68, 69\}$$

And the blue nodes

$$n_B = \{70, 71, 72, 73, 74, 75, 77, 84, 86, 87, 88, 90, 93, 94, 95, 96, 97, 98, 99\}.$$

As we can see, the inner core components $\{43, 46, 55\}$ and $\{84, 93, 99\}$ for $\epsilon = 1.351$ are nested in the inner core components for $\epsilon = 2.023$. But while $\{40, 59, 62, 64\}$ from the green outer core are completely nested in the larger inner core for $\epsilon = 2.023$, only 97 from the blue outer core $\{76, 97\}$ is now nested in the enlarged inner core.

B.2. Persistence in a Noisy Background

In Section 3.3.7, we analyzed the spectral sequences of two persistent clusters in backgrounds with different levels of noise. Instead of listing them, in this section we will visualize components of the Fiedler vector in the form of histograms to investigate how persistent the two clusters are. We start from the clean red and blue clusters (each having 50 data points). These are disjoint at $\epsilon = 0.656$ and $\epsilon = 1.309$, but become connected at $\epsilon =$

$1.962, 2.615, 3.267, 3.920$. As expected for the last four filtration parameters, the eigenvector $\vec{u}_0$ associated with $\lambda_0 = 0$ is a uniform superposition of all data points.

In Supplementary Figure B2, we show histograms of the Fiedler components, and the links between data points, at the different filtration parameter values. For $\epsilon = 1.962$ and $\epsilon = 2.615$, corresponding to $\lambda_1 = 0.622$ and $\lambda_1 = 9.963$ respectively, the two clusters are clearly identifiable based on the signs of their Fiedler components, but there are no obvious neck components with zero values. At $\epsilon = 2.615$, the neck connecting the two clusters becomes distinguishable as Fiedler components close to zero, and their associated data points are colored green or cyan. At $\epsilon = 3.267$, the neck becomes larger than the two clusters, and by $\epsilon = 3.920$, only a few points remain in the red and blue clusters. In this example, the distribution of Fiedler components changes from a bimodal distribution to a unimodal distribution as ϵ is increased. Tracking the changes in $\lambda_1 = 0.622 \rightarrow 9.963 \rightarrow 43.231 \rightarrow 76.940$, we see this change occurring where λ_1 changes most rapidly with ϵ.

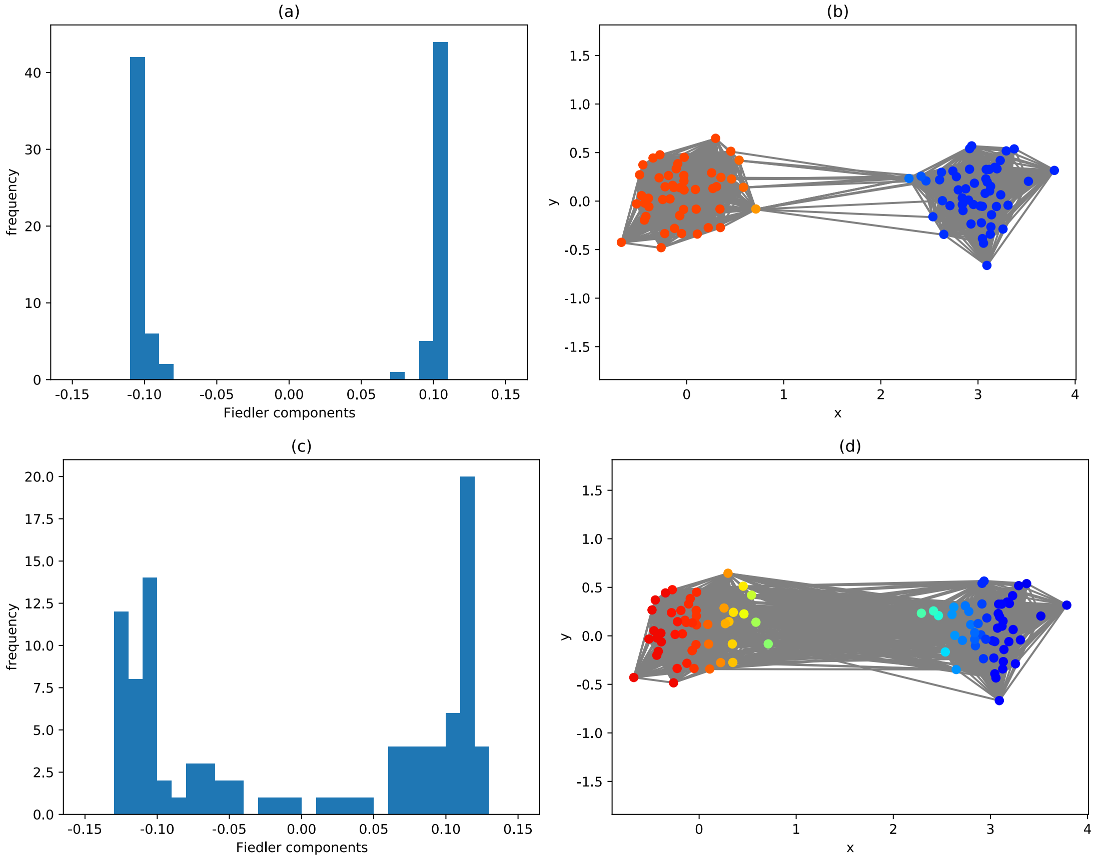

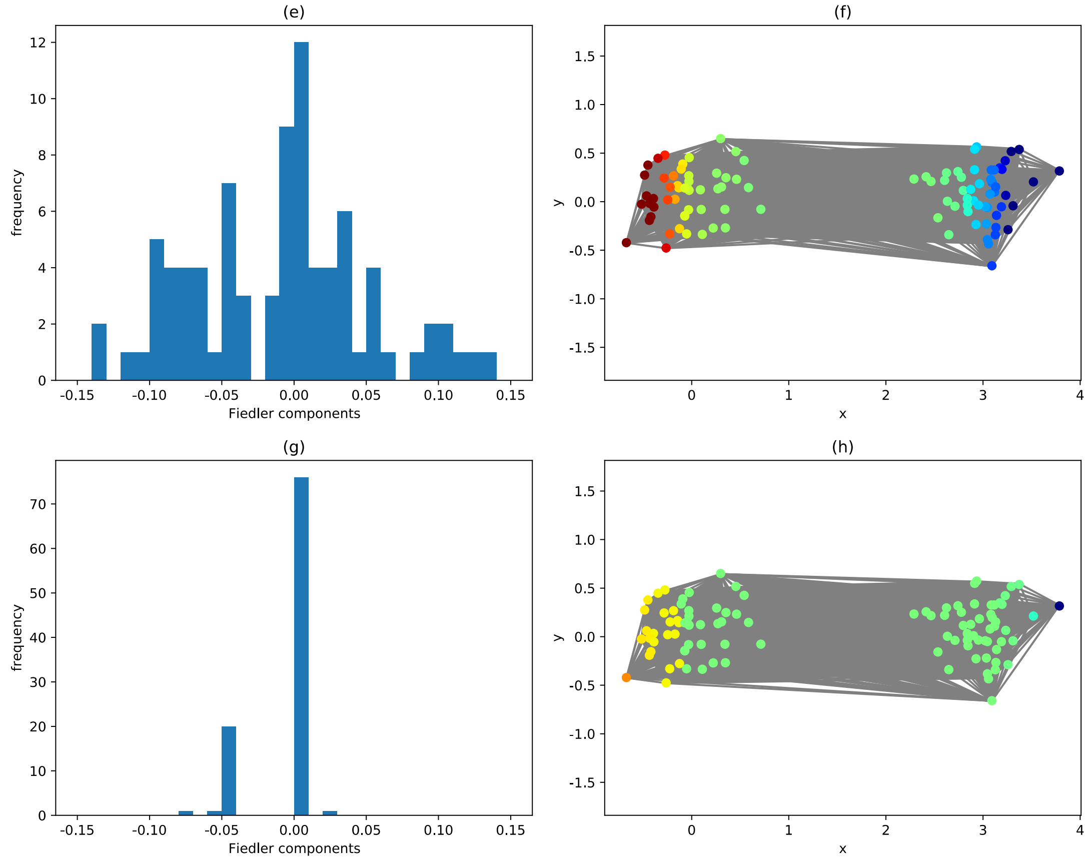

Figure B2: (left) Histograms of the Fiedler components, and (right) which of the data points in two 50-member clusters (red and blue) are linked at filtration parameter values of (a)-(b) $\epsilon = 1.962$, (c)-(d) $\epsilon = 2.615$, (e)-(f) $\epsilon = 3.267$, and (g)-(h) $\epsilon = 3.920$. In this figure, the data points are colored according to their Fiedler components, with red being close to -0.10, green being close to 0.00, and blue being close to $+0.10$.

Next, we considered the situation where the two 50-point clusters are in a noisy background with 30 dust particles shown in the rightmost panel of Supplementary Figure B2. At $\epsilon = 0.656$, we have 8 zero eigenvalues. One of the associated eigenvectors (zero eigenvectors) has nonzero components for the 50 red data points, along with 5 dust particles occurring within or close to the red cluster. We say therefore that this zero eigenvector represents the 50 red data points and 5 dust particles. Another zero eigenvector represents the 50 blue data points, along with 13 dust particles. The six remaining zero eigenvectors are various superpositions of the same 8 dust particles. From $\epsilon = 1.309$ to $\epsilon = 3.920$, $\lambda_0 = 0$ is non-degenerate, and as expected its eigenvector is the uniform superposition of all data points.

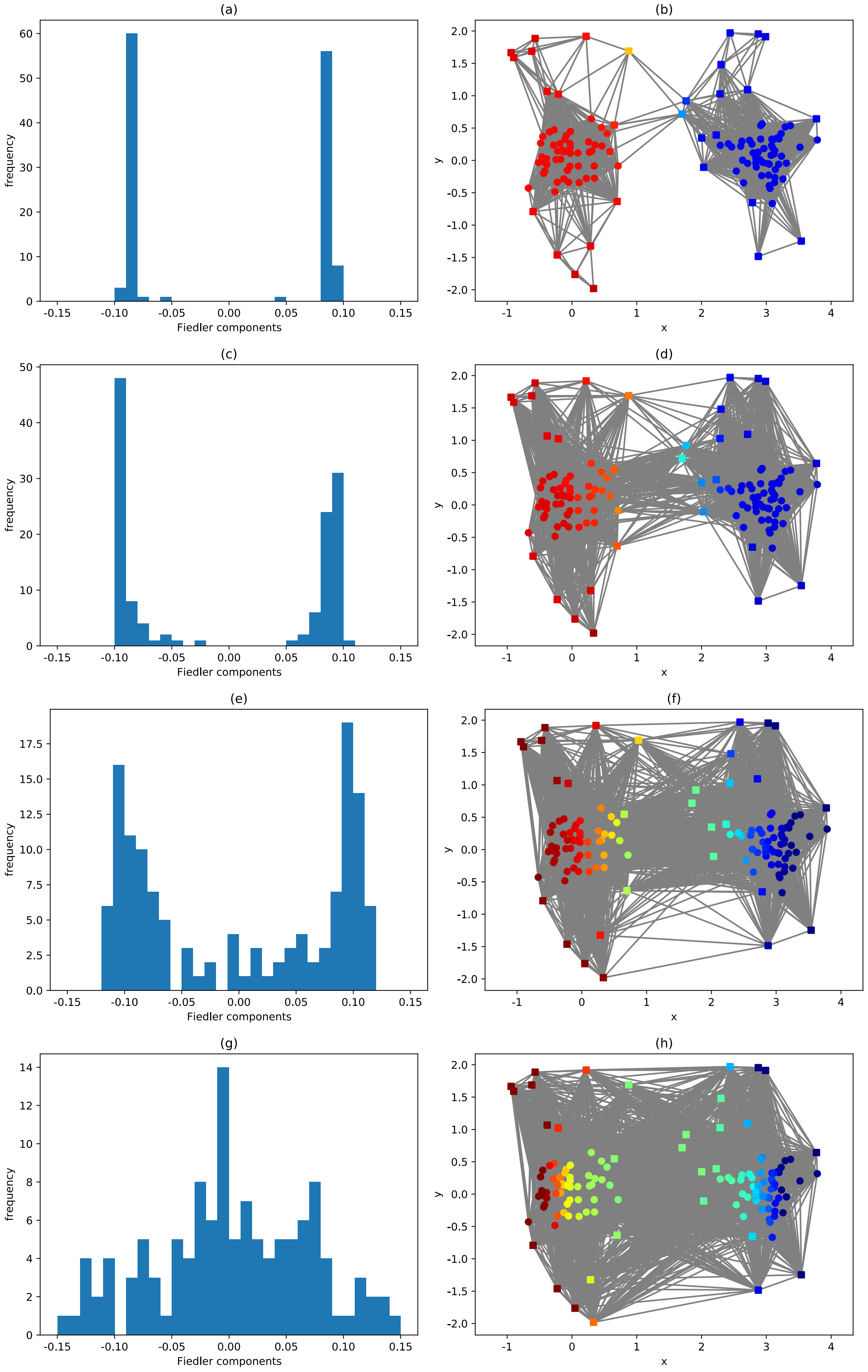
(a)
frequency
Fiedler components
(b)
y
x
(c)
frequency
Fiedler components
(d)
y
x
(e)
frequency
Fiedler components
(f)
y
x
(g)
frequency
Fiedler components
(h)
y
x

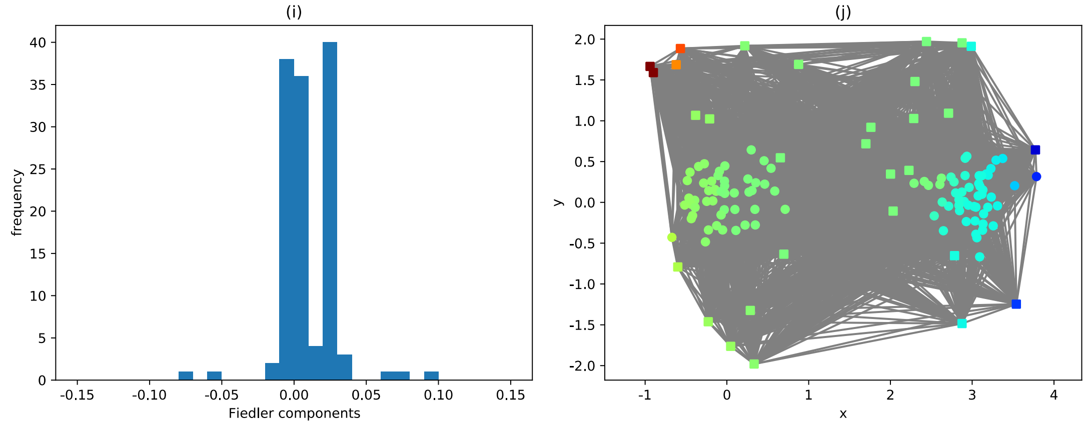

Figure B3: (left) Histograms of the Fiedler components, and (right) which of the data points in two 50-member clusters (red and blue) or 30 dust particles in the noisy background are linked at filtration parameter values of (a)-(b) $\epsilon = 1.30892$, (c)-(d) $\epsilon = 1.96173$, (e)-(f) $\epsilon = 2.61453$, (g)-(h) $\epsilon = 3.26734$, and (i)-(j) $\epsilon = 3.92015$. In this figure, the data points are colored according to their Fiedler components, with red being around -0.10, green being around 0.00, and blue being around $+0.10$. Moreover, data points belonging to the two clean clusters are shown as circles, whereas dust particles are shown as squares.

To compare the noisy and the clean situations, we show in Supplementary Figure B3 histograms of the Fiedler components, and the links between data points, at the different filtration parameter values. For $\epsilon = 1.309, 1.962, 2.615$, corresponding to $\lambda_1 = 0.183, 0.622, 9.963$ respectively, the two clusters are clearly identifiable based on their Fiedler components. As early as $\epsilon = 1.309$, two data points belonging to the neck can be identified. In Supplementary Figure B3(b), one is colored yellow, and the other cyan. Interestingly, both are dust particles that have been incorporated into the red and blue clusters. As ϵ increases, the neck linking the two clusters becomes more prominent, and an increasing number of data points are colored green or cyan. Eventually, most data points become part of the neck, and a small number of points remain for the two original clusters. As the above is happening, the distribution of Fiedler components also changes from bimodal to unimodal. The crossover occurs at the point where λ_1 changes most rapidly with ϵ. This progression is the same as for the clean situation.

The main notable differences between the clean and noisy situations are as follows. First, the clusters in the clean situation are compact, close to circular, and remain at their maximum sizes over a broad range of ϵ. In the noisy situation, the clusters continue to grow with ϵ, by incorporating more and more dust particles. Because the dust particles appear in a background with a different density compared to the clusters, the clusters appear as if they are growing spikes as they absorb dust particles. This feature leads to the more important difference between the two situations, i.e., in the clean situation, a *direct* neck forms between the parts of the clusters closest to each other. As ϵ increases, this direct neck becomes fatter, but otherwise the same topologically. In the noisy situation, however, as ϵ increases, in addition to the formation of a direct neck, *indirect* necks also form between the clusters between spikes that are pointing toward each other, and thus always involve dust particles. The formation of direct and indirect necks between the two clusters lead to complex topologies in the region between them.

C. Spectral Sequences for Mar 2020 TWSE Crash

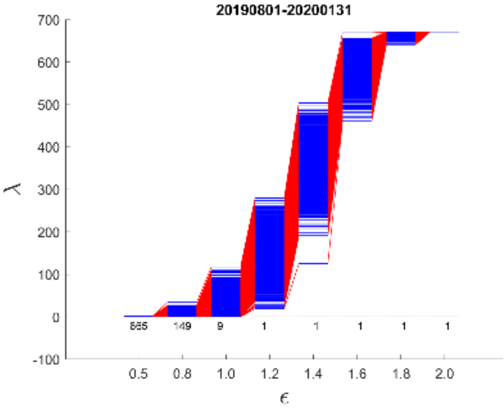

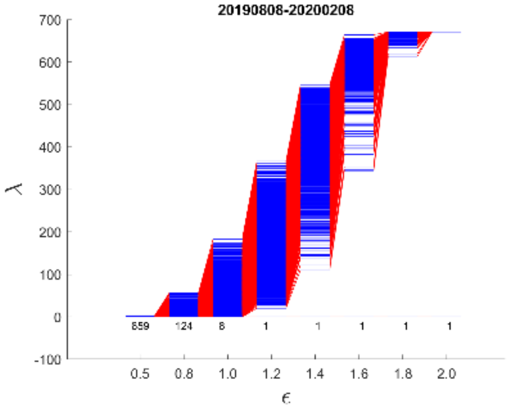

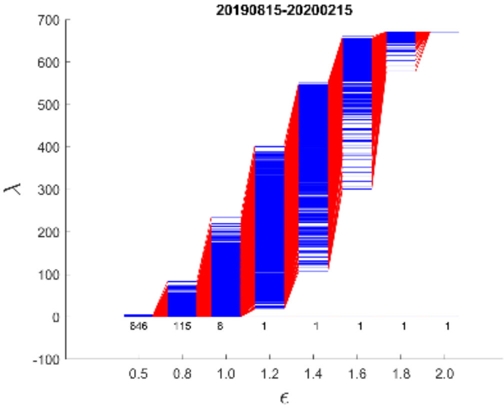

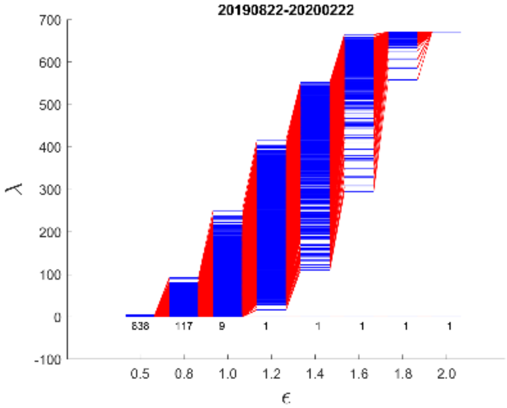

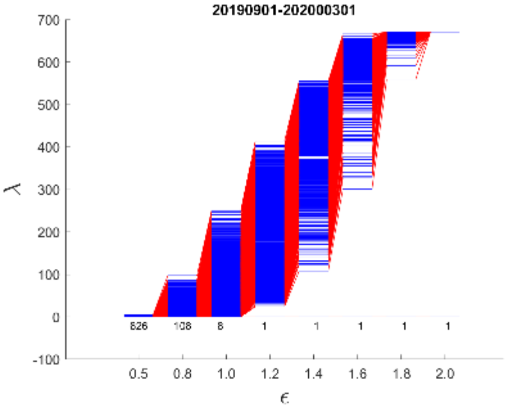

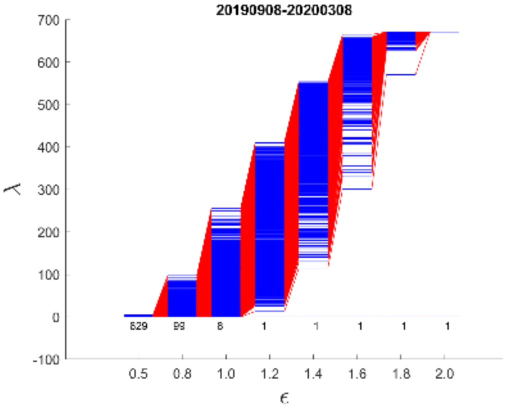

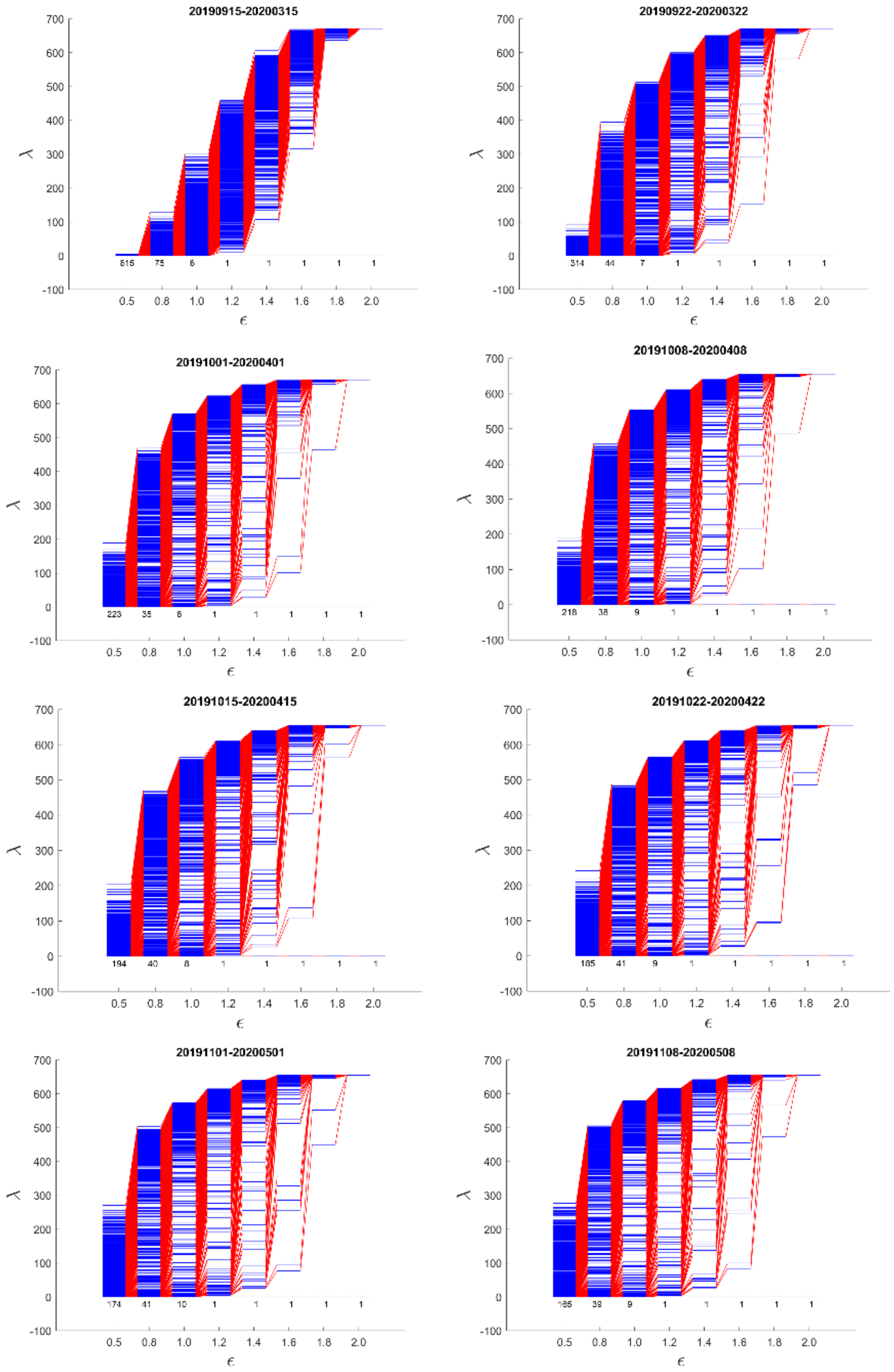
20190915-20200315
20190922-20200322
20191001-20200401
20191008-20200408
20191015-20200415
20191022-20200422
20191101-20200501
20191108-20200508

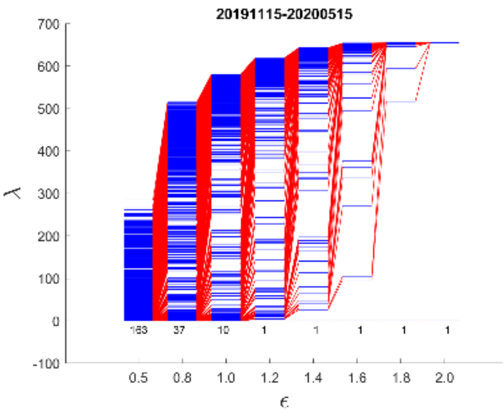
20191115-20200515

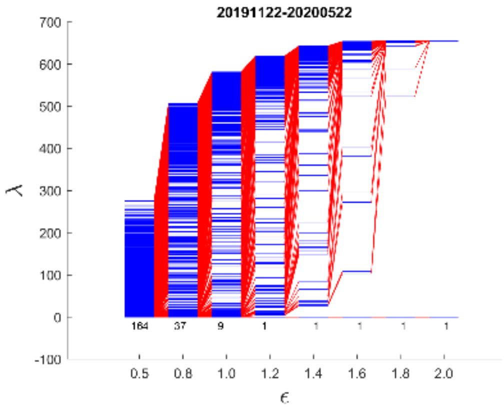
20191122-20200522

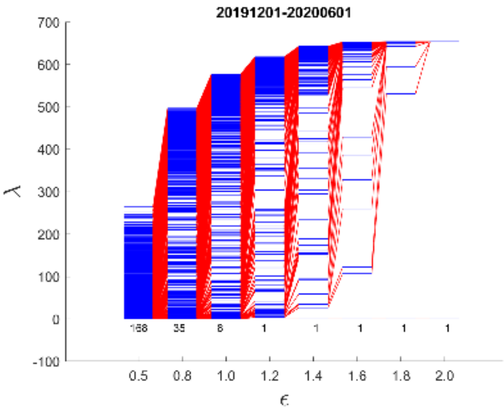
20191201-20200601

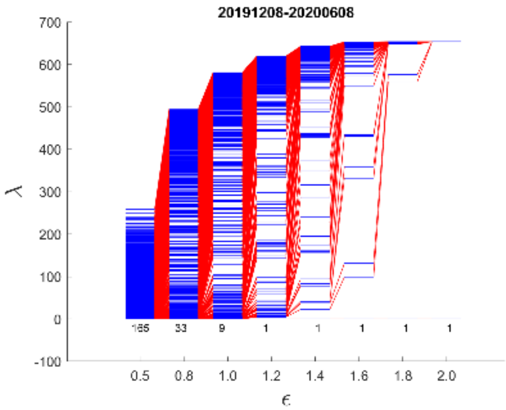
20191208-20200608

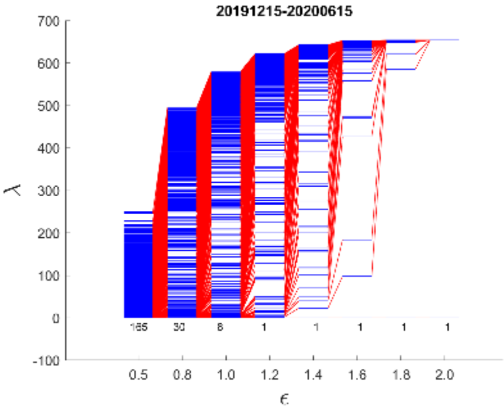
20191215-20200615

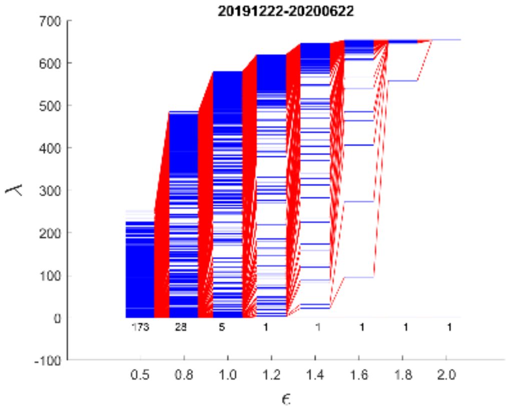
20191222-20200622

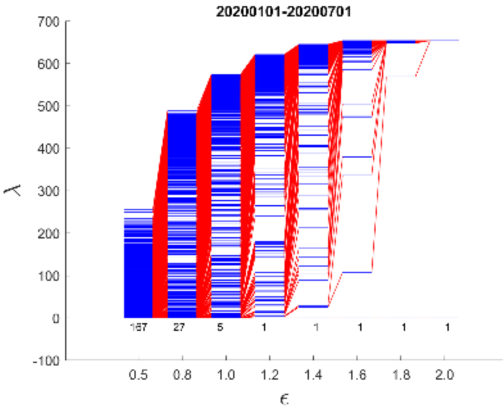
20200101-20200701

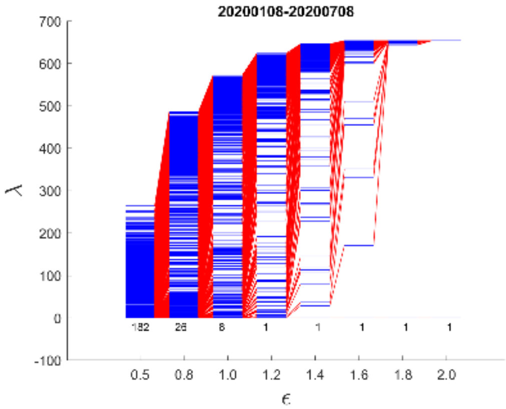
20200108-20200708

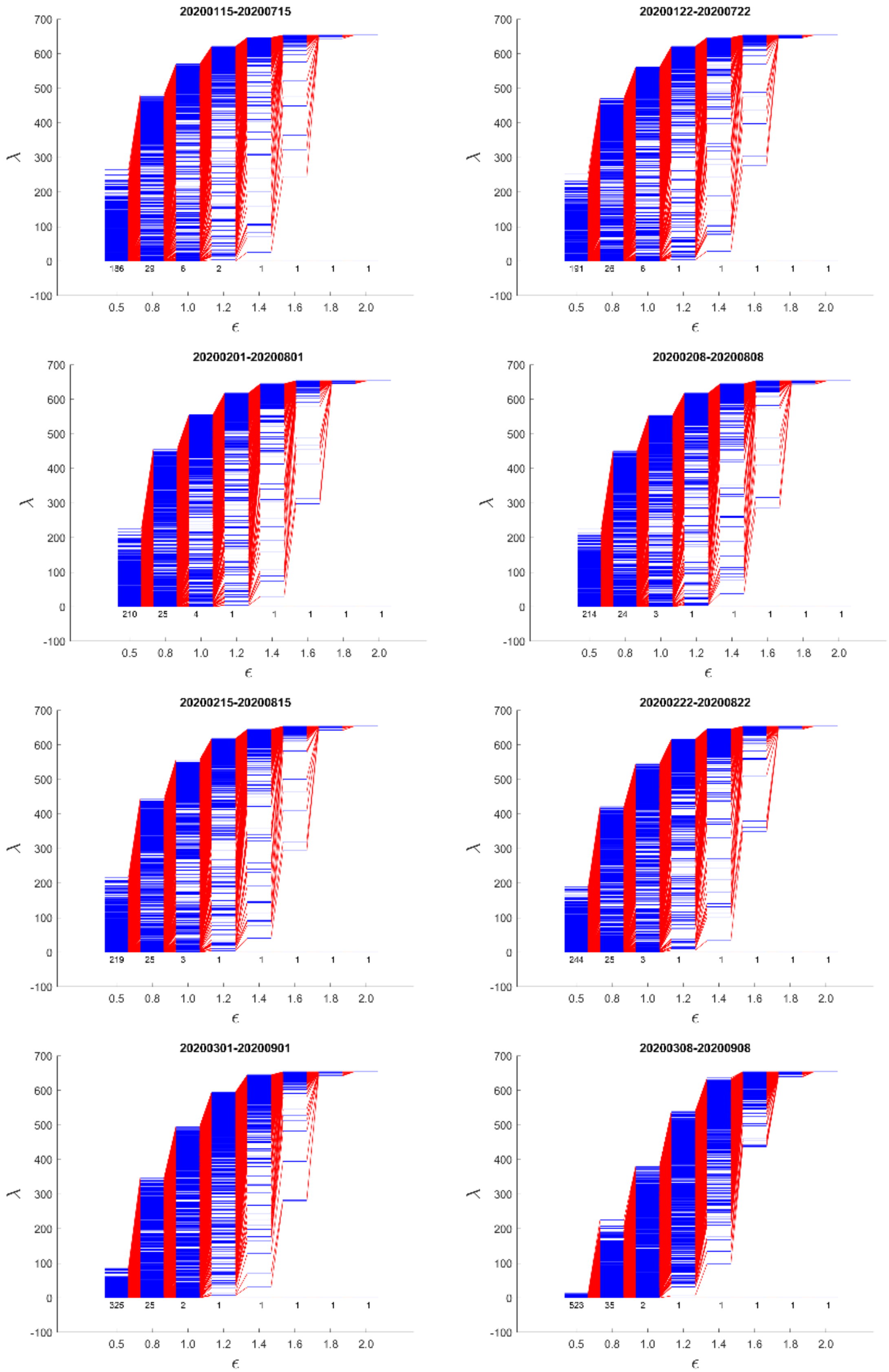

20200115-20200715
20200122-20200722
20200201-20200801
20200208-20200808
20200215-20200815
20200222-20200822
20200301-20200901
20200308-20200908

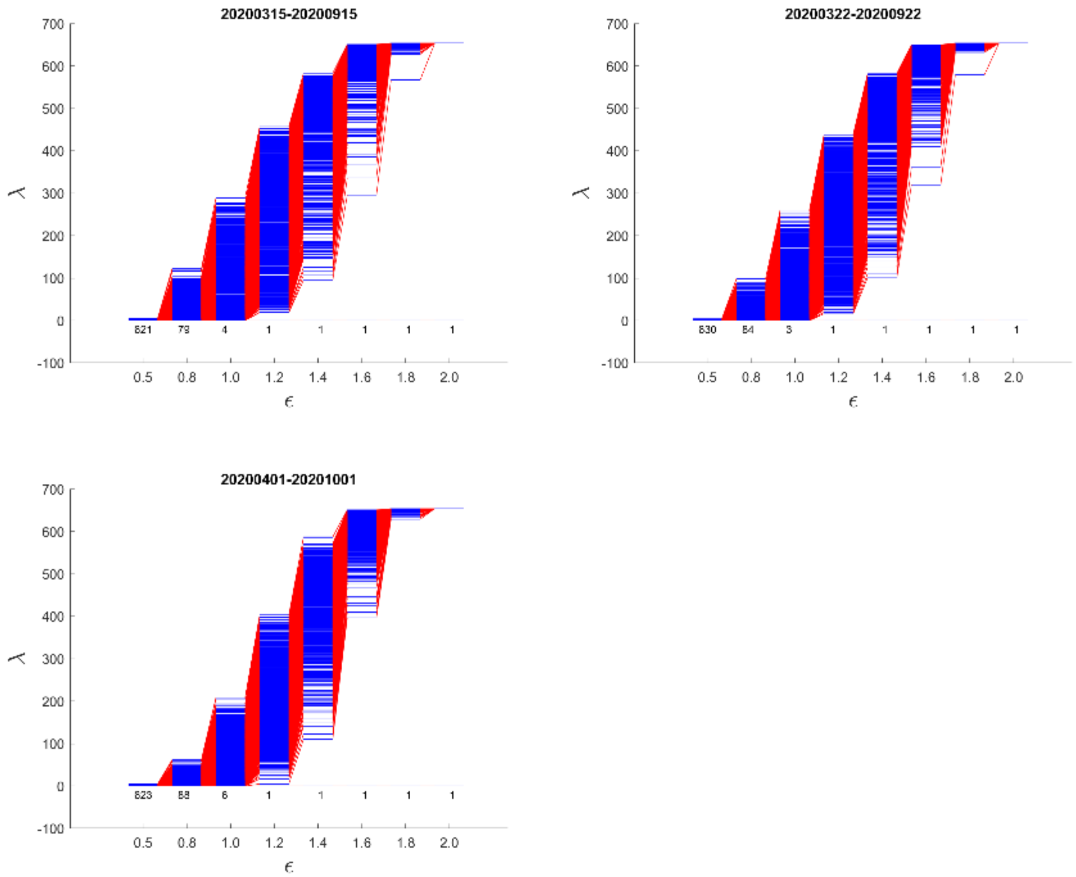

Figure C1. Filtration parameter ϵ versus eigenvalues. We studied them at eight prescribed ϵ values, namely ϵ = 0.5, 0.8, 1.0, 1.2, and 1.4, 1.6, 1.8, and 2.0, respectively. The market data are collected from TWSE during 1 Aug 2019 to 30 Sep 2020, which covers the Mar 2020 TWSE COVID-19 crash. The window size is six months. In the subfigures, the time window is indicated as *YYYYMMDD* of the starting date to *YYYYMMDD* of the ending date.

D. Spectral Sequences for Sep 2018 TWSE Mini-Crash

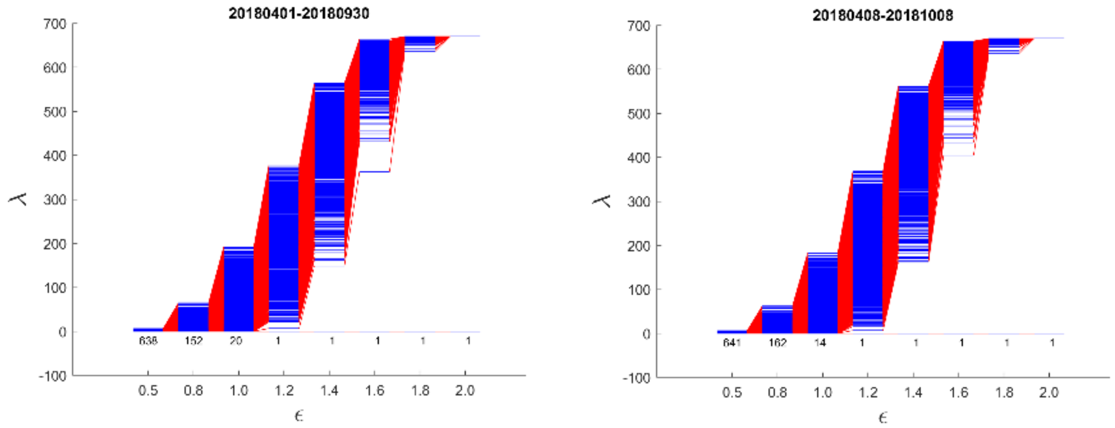

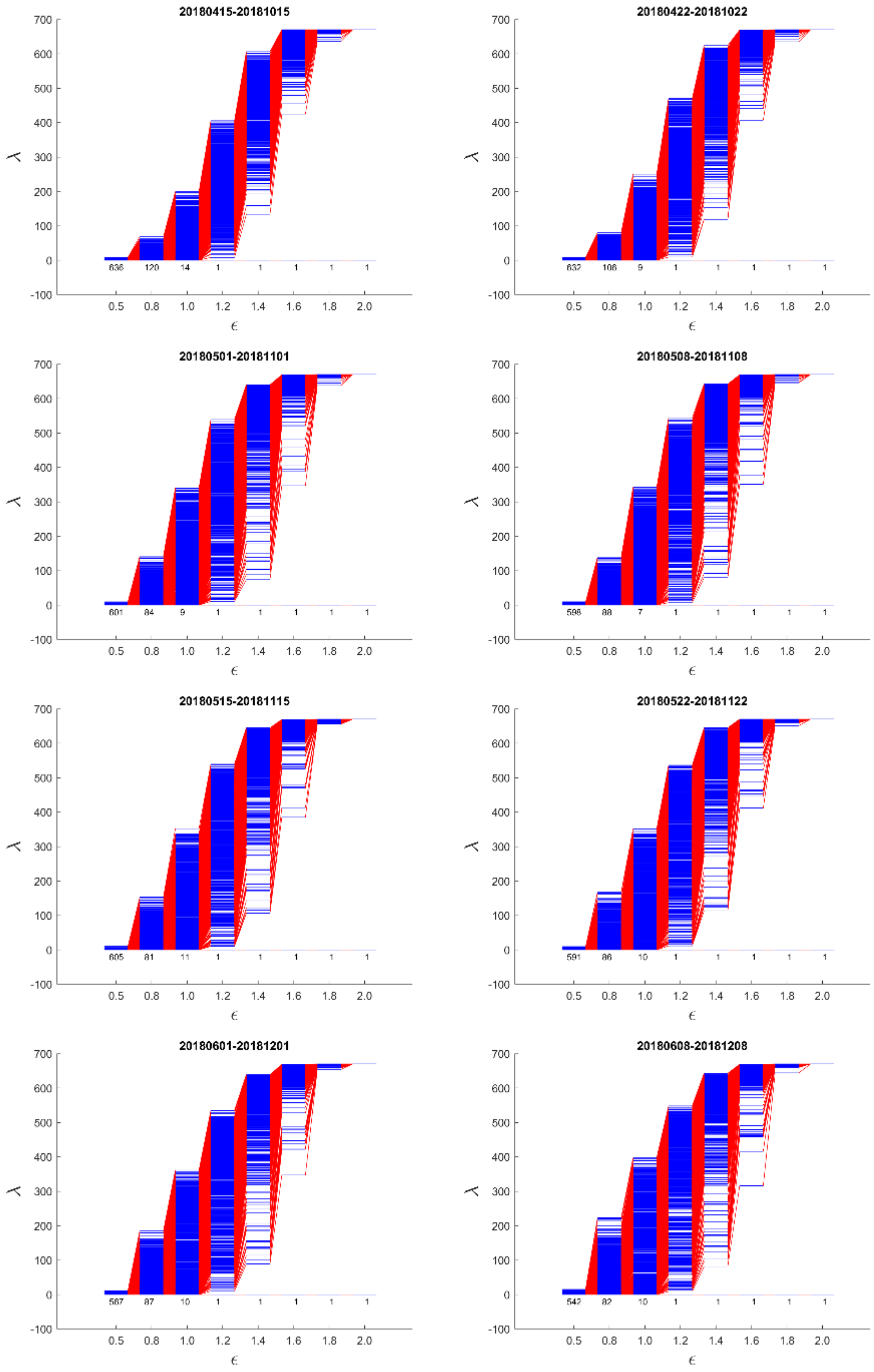
20180415-20181015
636 120 14 1 1 1 1 1
20180422-20181022
632 106 9 1 1 1 1 1
20180501-20181101
601 84 9 1 1 1 1 1
20180508-20181108
598 88 7 1 1 1 1 1
20180515-20181115
605 81 11 1 1 1 1 1
20180522-20181122
591 86 10 1 1 1 1 1
20180601-20181201
567 87 10 1 1 1 1 1
20180608-20181208
542 82 10 1 1 1 1 1
λ
700 600 500 400 300 200 100 0 -100
0.5 0.8 1.0 1.2 1.4 1.6 1.8 2.0
ϵ

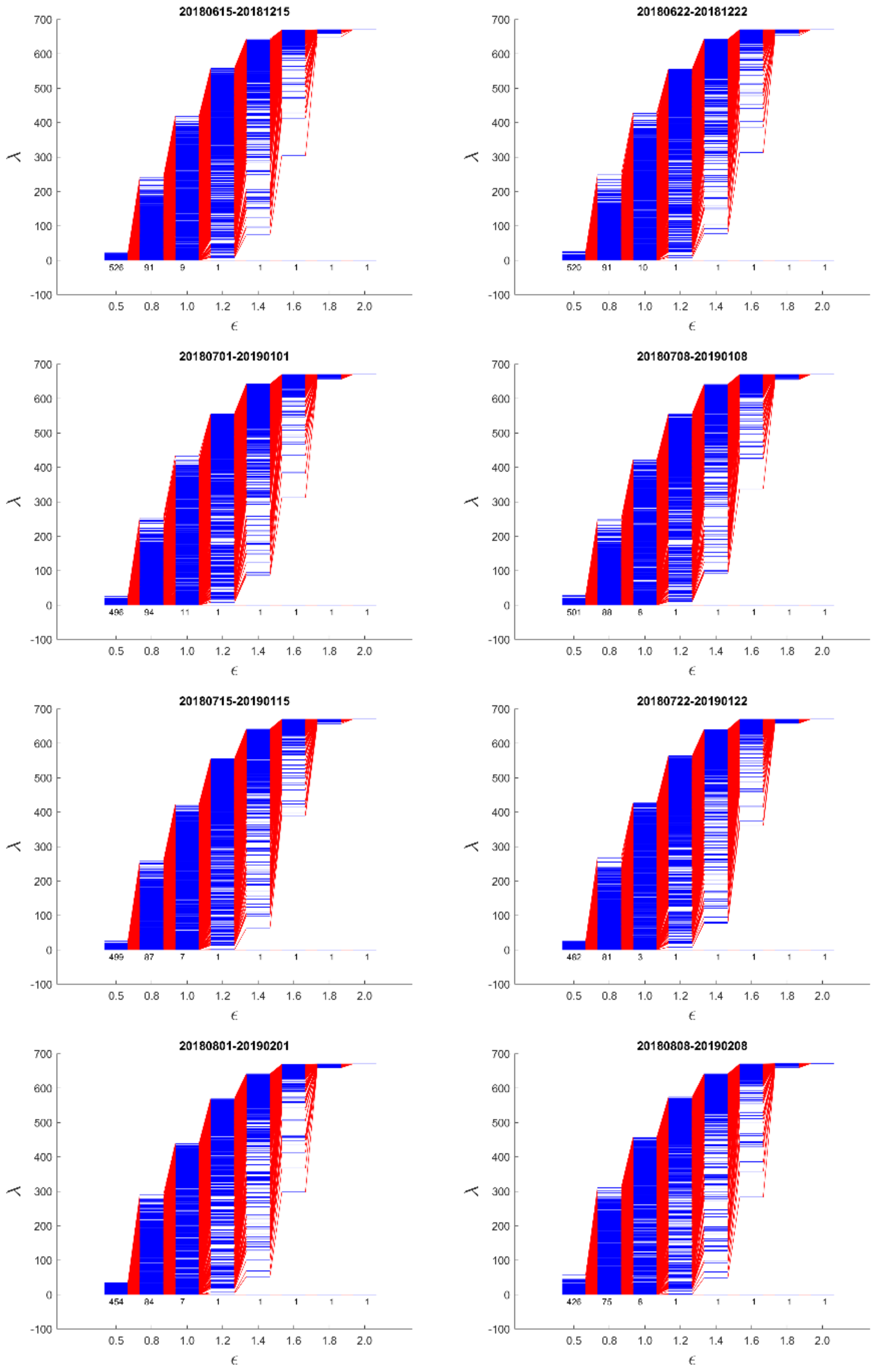
20180615-20181215
20180622-20181222
20180701-20190101
20180708-20190108
20180715-20190115
20180722-20190122
20180801-20190201
20180808-20190208
λ
ϵ

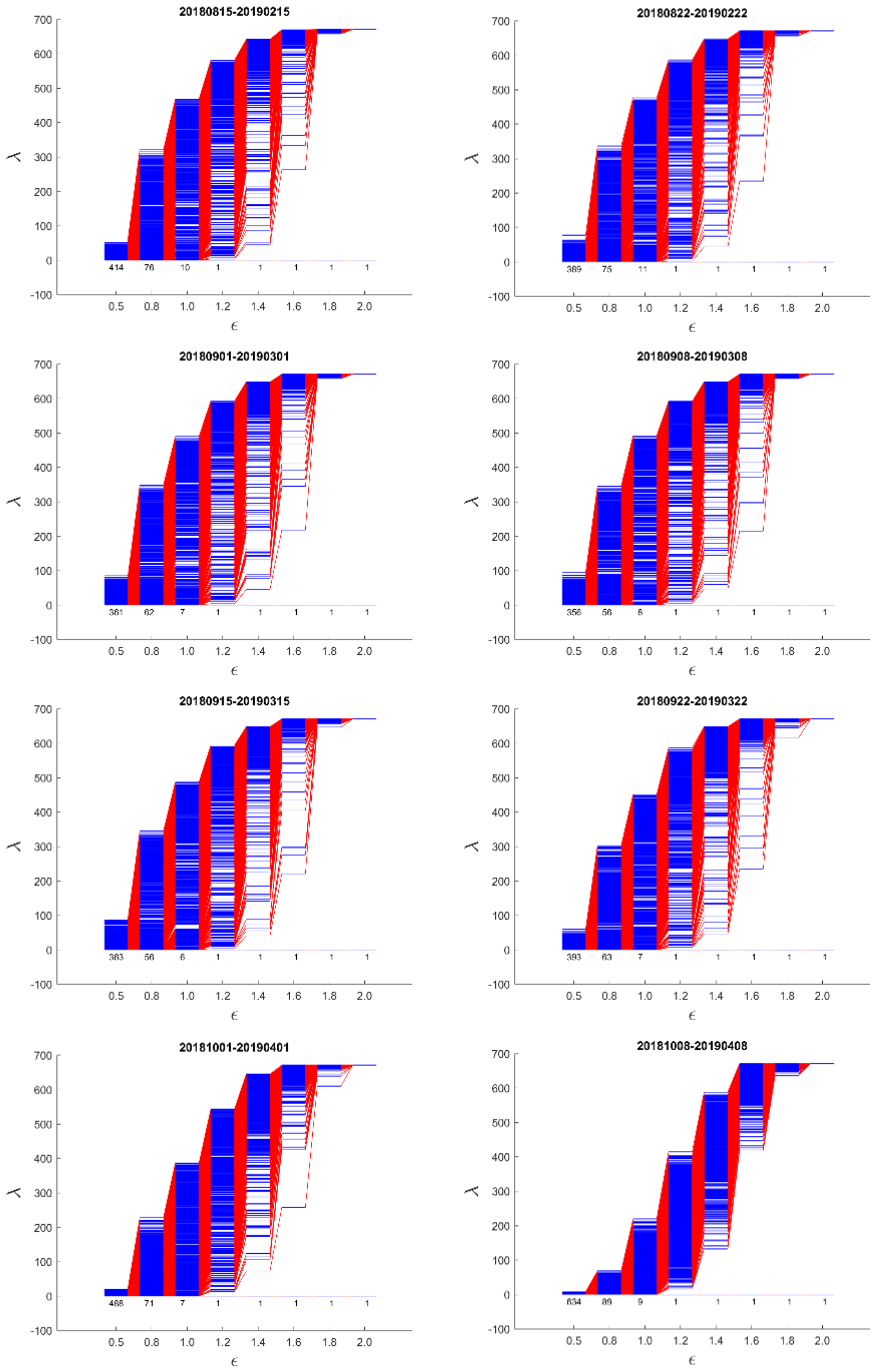
20180815-20190215
20180822-20190222
20180901-20190301
20180908-20190308
20180915-20190315
20180922-20190322
20181001-20190401
20181008-20190408
λ
ϵ
700
600
500
400
300
200
100
0
-100
0.5
0.8
1.0
1.2
1.4
1.6
1.8
2.0

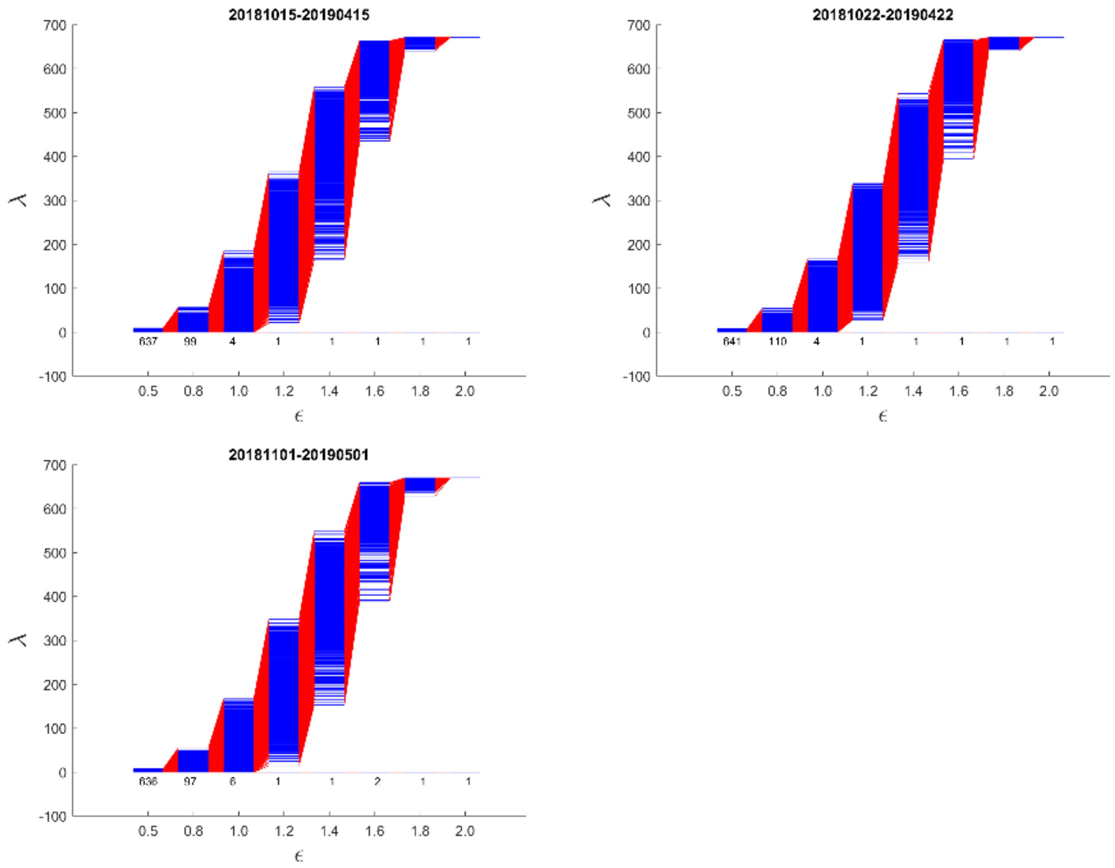

Figure D1. Filtration parameter ϵ versus eigenvalues. We studied them at eight prescribed ϵ values, namely ϵ = 0.5, 0.8, 1.0, 1.2, and 1.4, 1.6, 1.8, and 2.0, respectively. The market data are collected from TWSE during 1 Apr 2018 to 30 Apr 2019, which covers the Sep 2018 mini crash. The window size is six months. In the subfigures, the time window is indicated as *YYYYMMDD* of the starting date to *YYYYMMDD* of the ending date.

E. Spectral Sequences for Mar 2020 SGX Crash

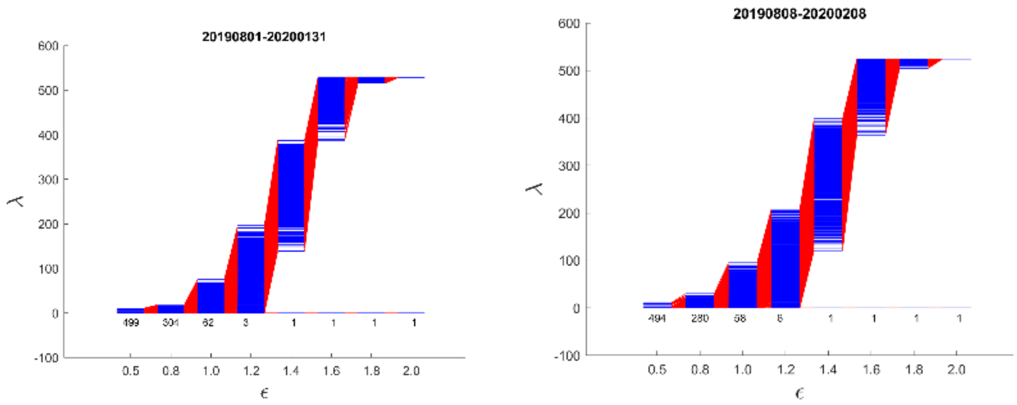

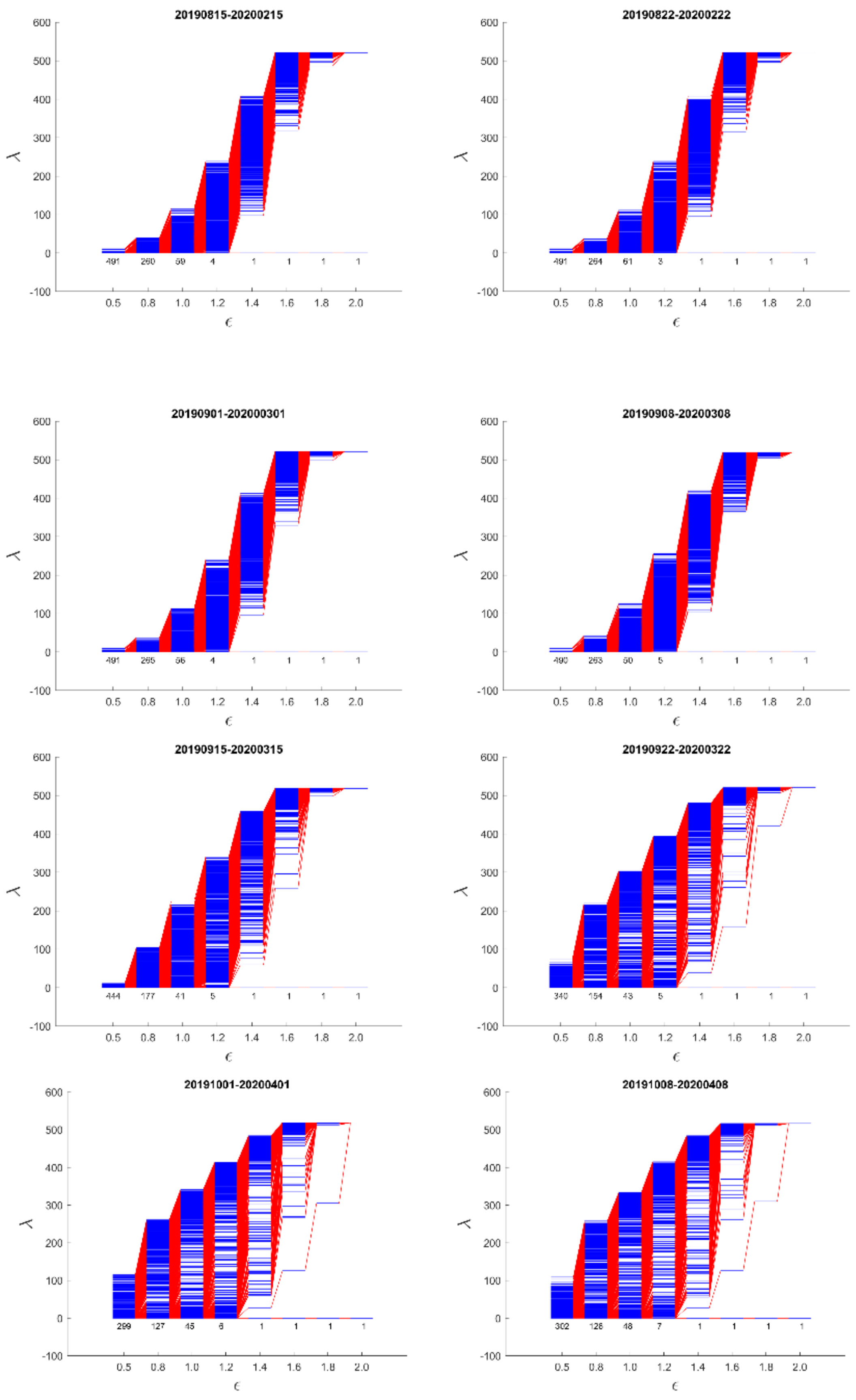
20190815-20200215
20190822-20200222
20190901-202000301
20190908-20200308
20190915-20200315
20190922-20200322
20191001-20200401
20191008-20200408
λ
ϵ

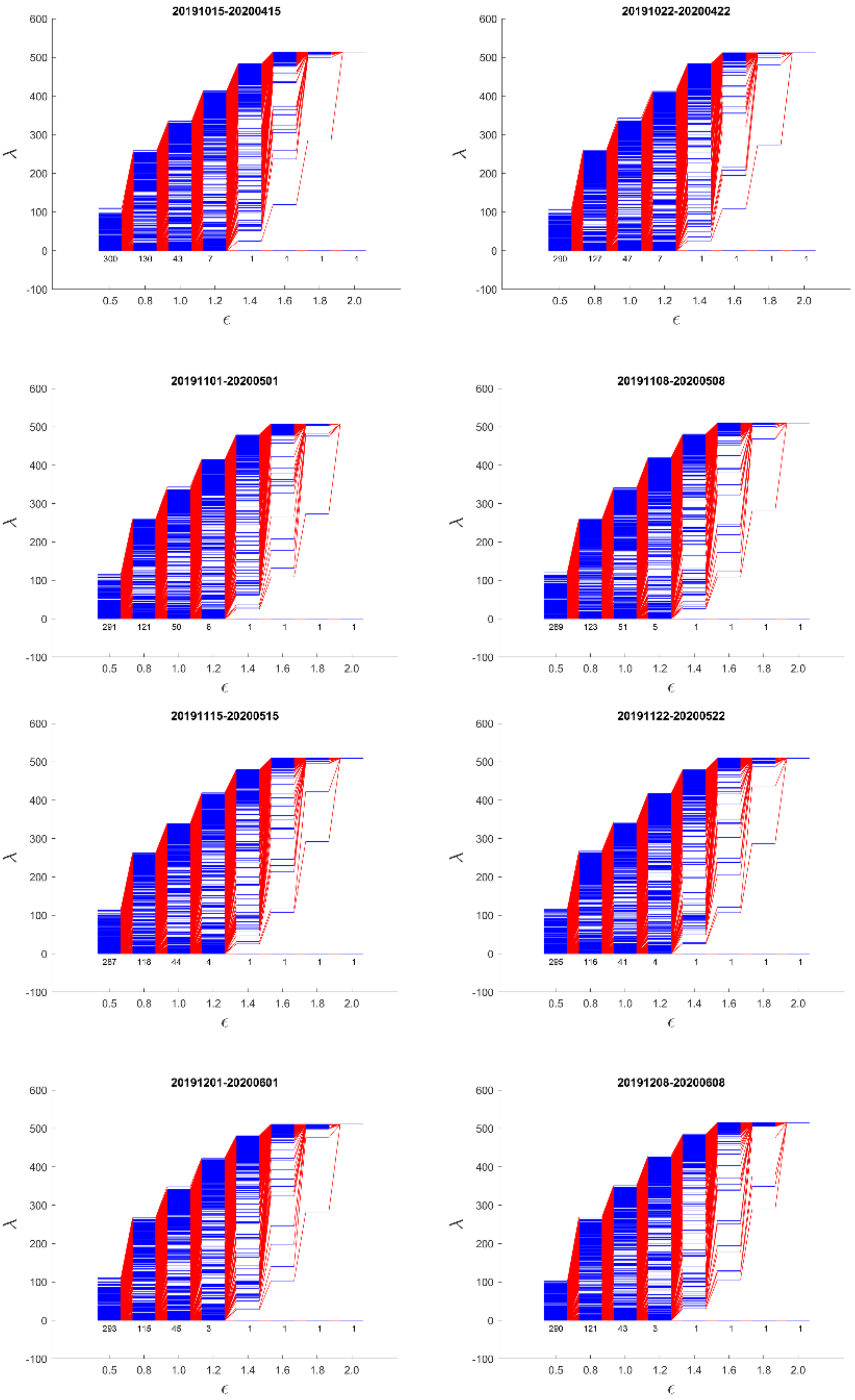
20191015-20200415
20191022-20200422
20191101-20200501
20191108-20200508
20191115-20200515
20191122-20200522
20191201-20200601
20191208-20200608

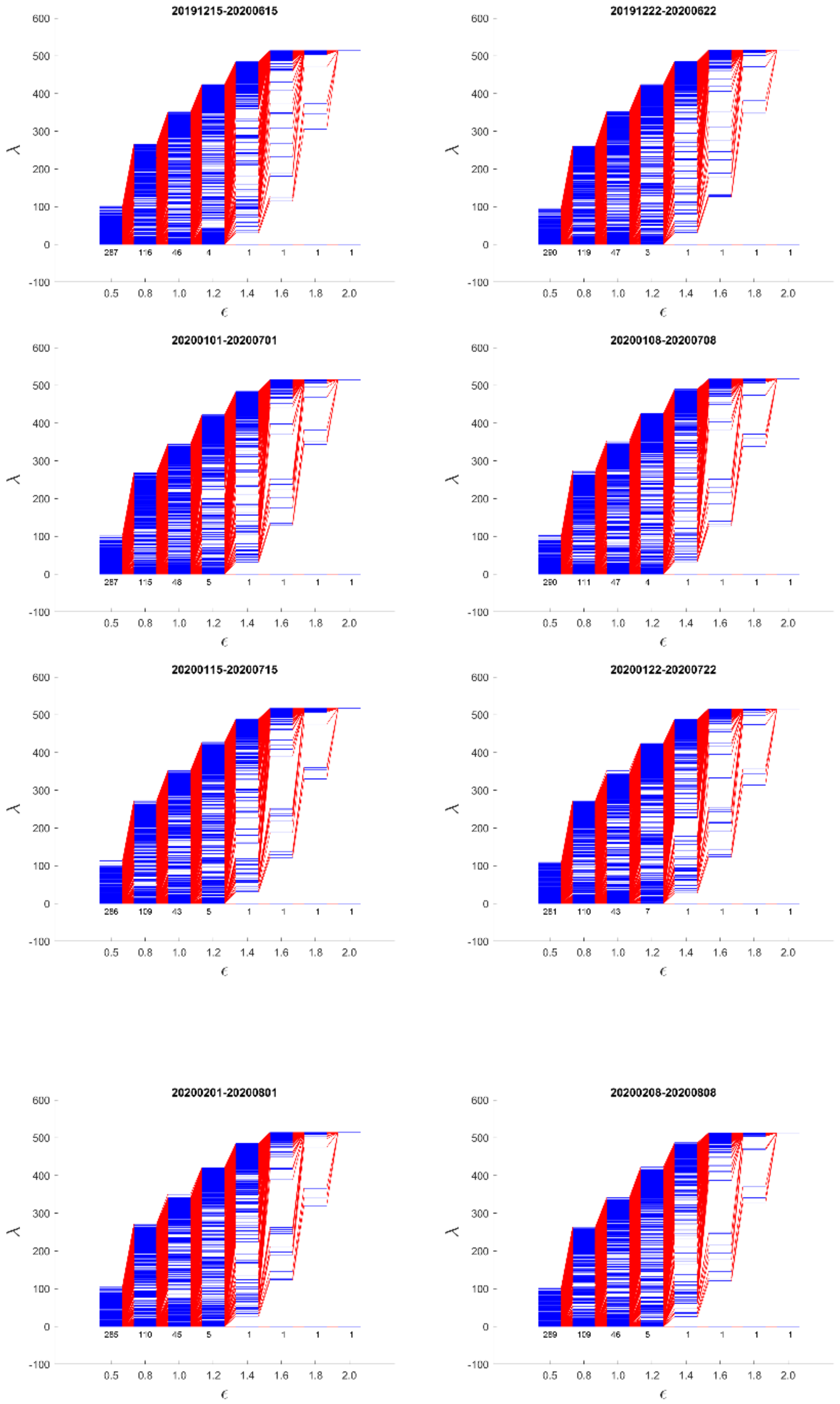
20191215-20200615
20191222-20200622
20200101-20200701
20200108-20200708
20200115-20200715
20200122-20200722
20200201-20200801
20200208-20200808
λ
ϵ

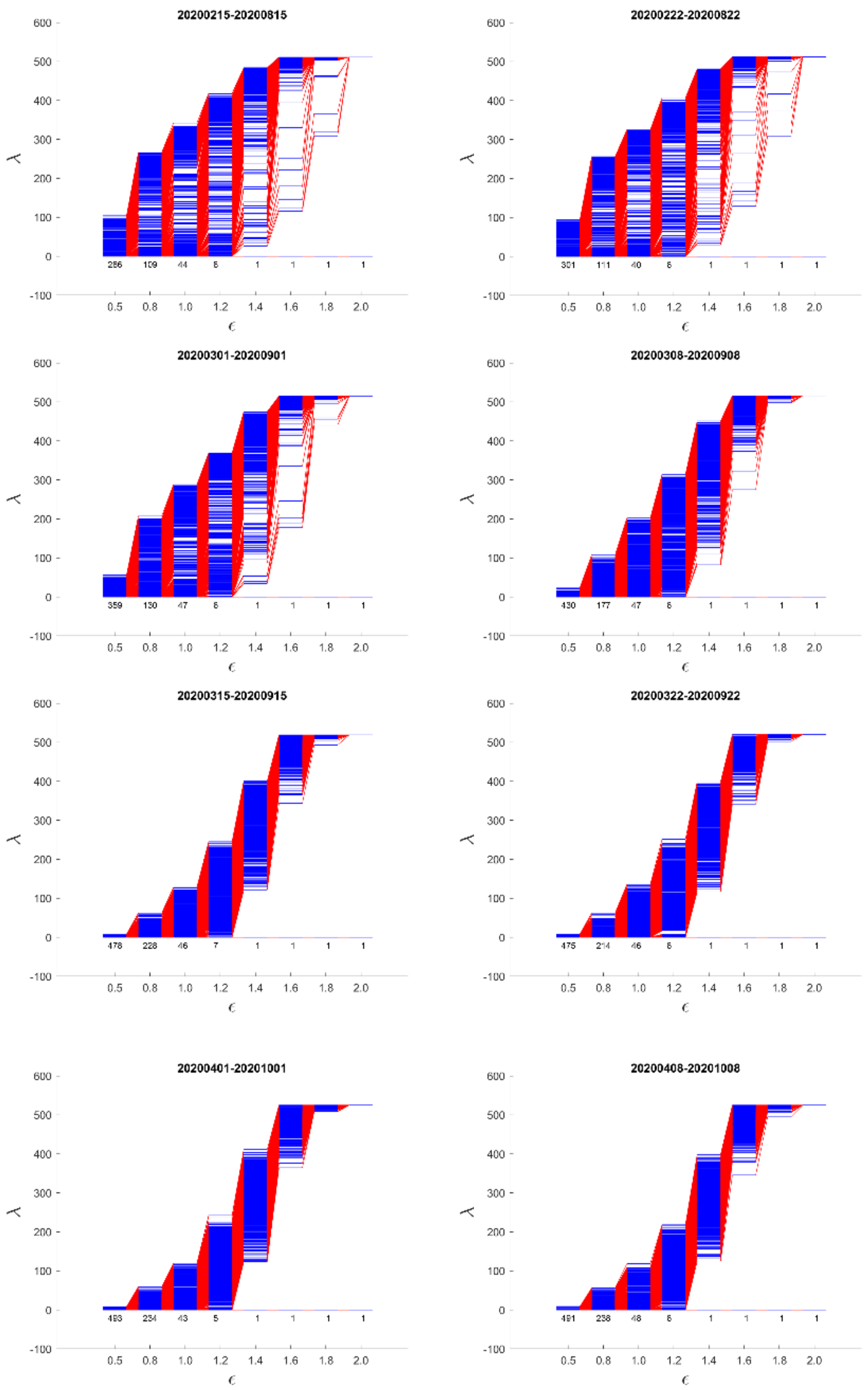
20200215-20200815
286 109 44 8 1 1 1 1
20200222-20200822
301 111 40 8 1 1 1 1
20200301-20200901
359 130 47 8 1 1 1 1
20200308-20200908
430 177 47 8 1 1 1 1
20200315-20200915
478 228 46 7 1 1 1 1
20200322-20200922
475 214 46 8 1 1 1 1
20200401-20201001
493 234 43 5 1 1 1 1
20200408-20201008
491 238 48 8 1 1 1 1
λ
ϵ
600 500 400 300 200 100 0 -100
0.5 0.8 1.0 1.2 1.4 1.6 1.8 2.0

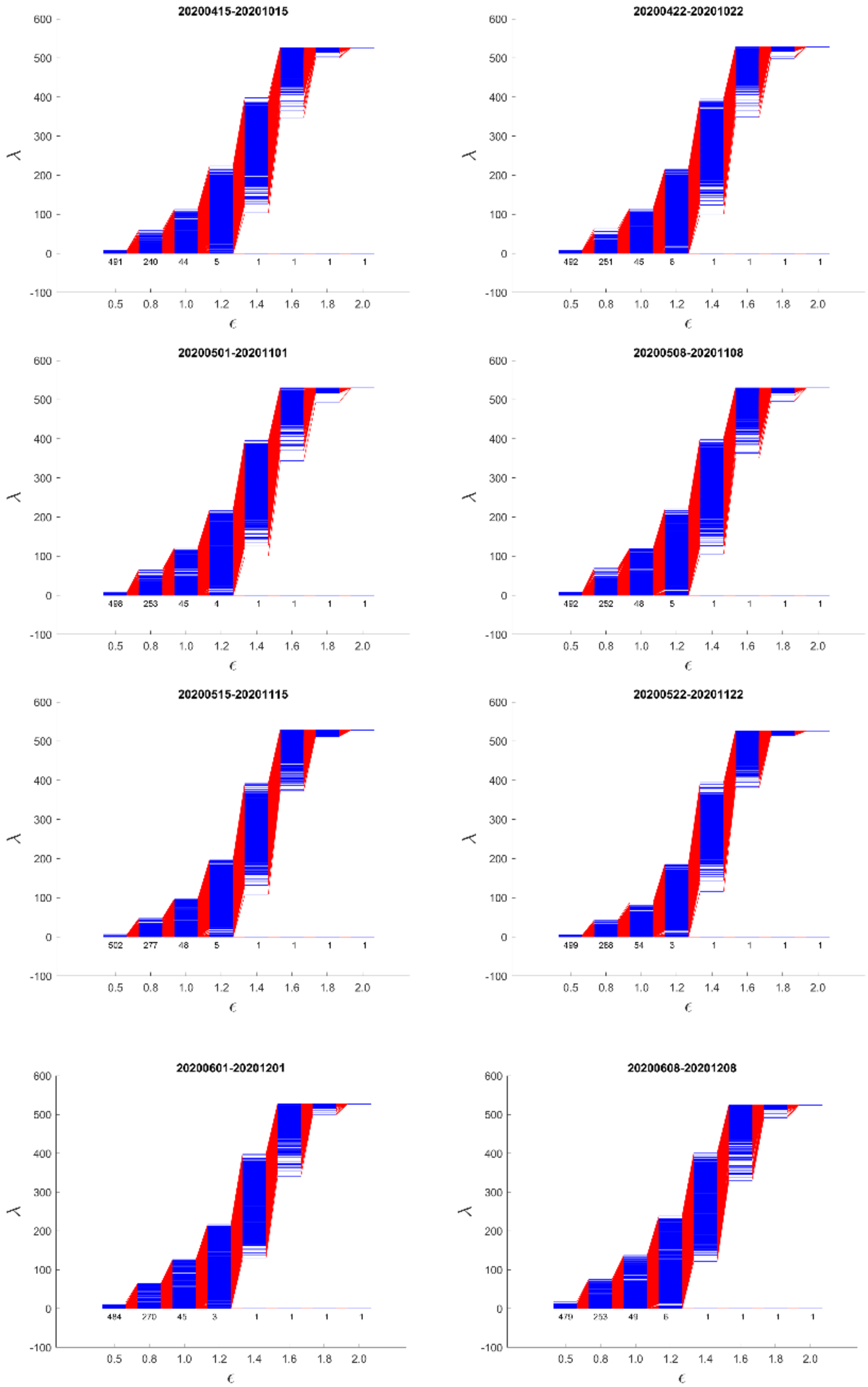
20200415-20201015
491 240 44 5 1 1 1 1
20200422-20201022
492 251 45 6 1 1 1 1
20200501-20201101
498 253 45 4 1 1 1 1
20200508-20201108
492 252 48 5 1 1 1 1
20200515-20201115
502 277 48 5 1 1 1 1
20200522-20201122
499 288 54 3 1 1 1 1
20200601-20201201
484 270 45 3 1 1 1 1
20200608-20201208
479 253 49 6 1 1 1 1
λ
ϵ
0.5 0.8 1.0 1.2 1.4 1.6 1.8 2.0
-100 0 100 200 300 400 500 600

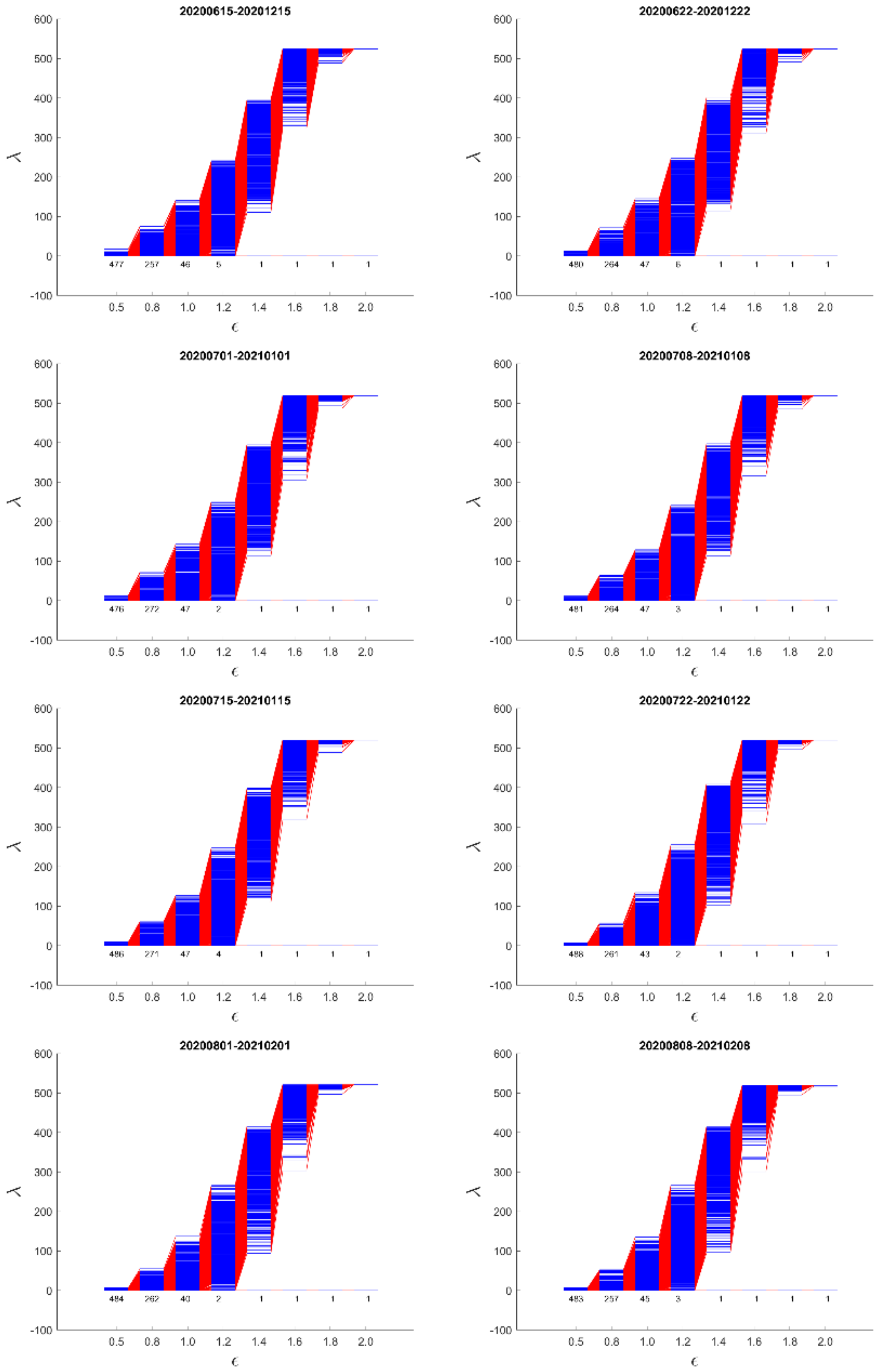
20200615-20201215
477 257 46 5 1 1 1 1
20200622-20201222
480 264 47 6 1 1 1 1
20200701-20210101
476 272 47 2 1 1 1 1
20200708-20210108
481 264 47 3 1 1 1 1
20200715-20210115
486 271 47 4 1 1 1 1
20200722-20210122
488 261 43 2 1 1 1 1
20200801-20210201
484 262 40 2 1 1 1 1
20200808-20210208
483 257 45 3 1 1 1 1
λ
ϵ
600 500 400 300 200 100 0 -100
0.5 0.8 1.0 1.2 1.4 1.6 1.8 2.0

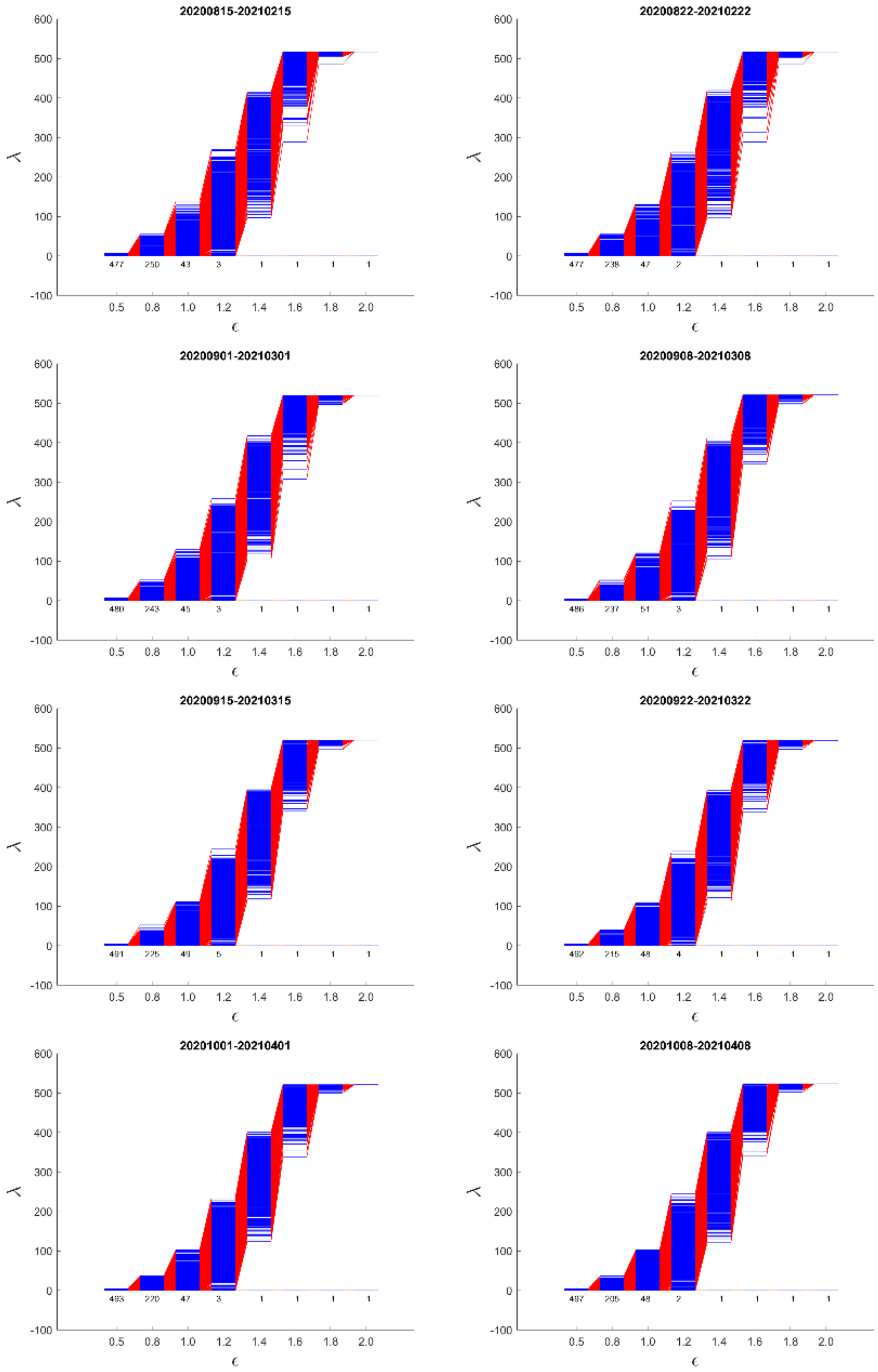

20200815-20210215
20200822-20210222
20200901-20210301
20200908-20210308
20200915-20210315
20200922-20210322
20201001-20210401
20201008-20210408
λ
ϵ

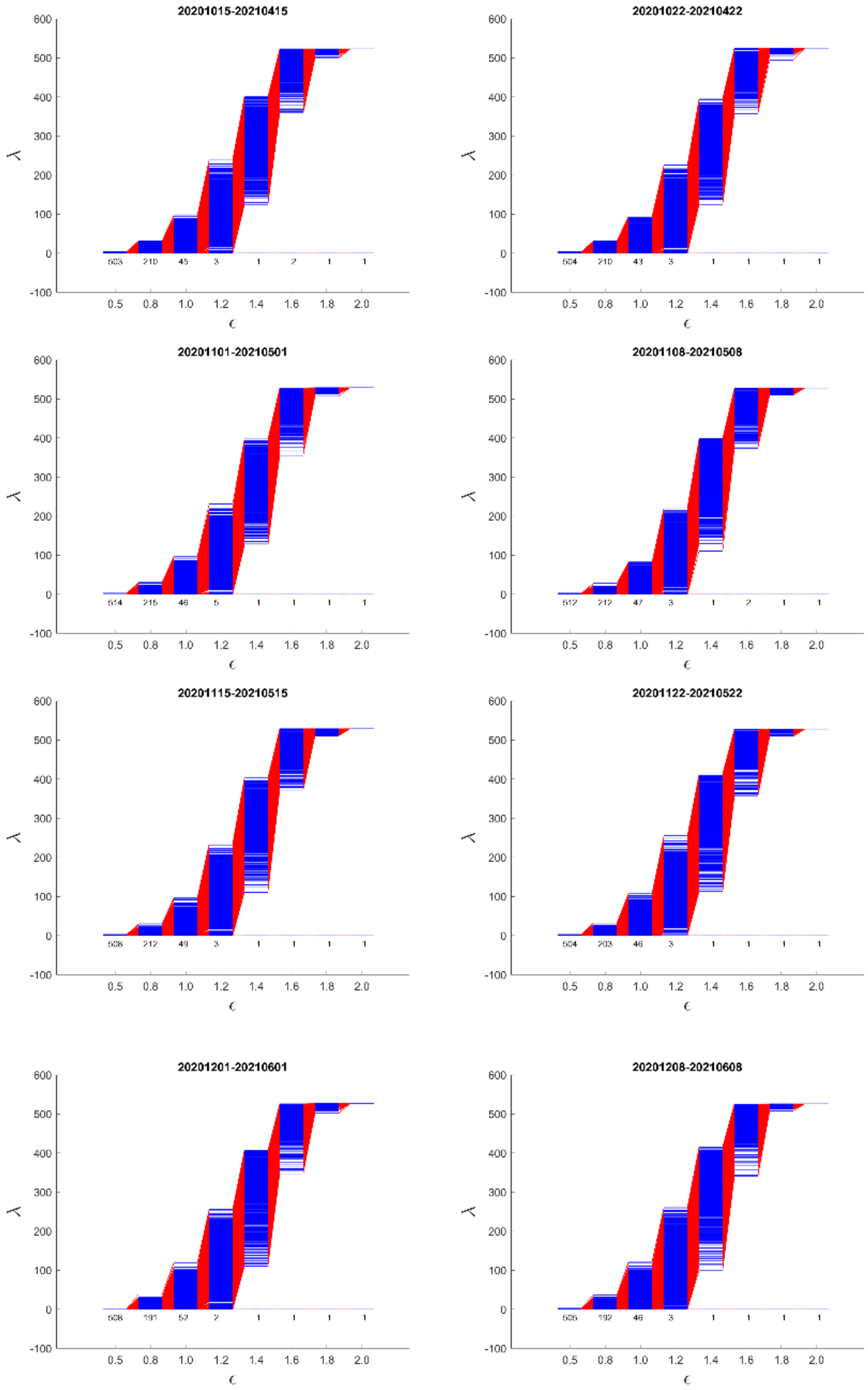
20201015-20210415
503 210 45 3 1 2 1 1
20201022-20210422
504 210 43 3 1 1 1 1
20201101-20210501
514 215 46 5 1 1 1 1
20201108-20210508
512 212 47 3 1 2 1 1
20201115-20210515
508 212 49 3 1 1 1 1
20201122-20210522
504 203 46 3 1 1 1 1
20201201-20210601
508 191 52 2 1 1 1 1
20201208-20210608
505 192 46 3 1 1 1 1
λ
600 500 400 300 200 100 0 -100
0.5 0.8 1.0 1.2 1.4 1.6 1.8 2.0
∈

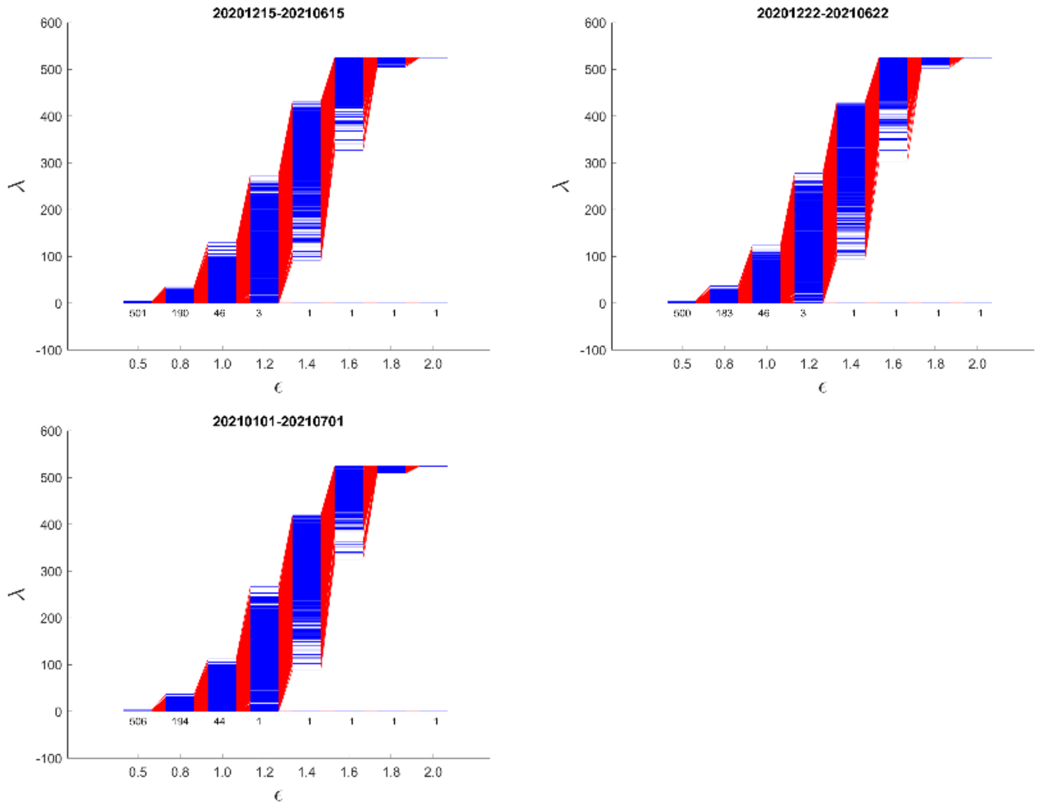

Figure E1: Index versus eigenvalues. We studied them at eight prescribed ϵ values, namely ϵ = 0.5, 0.8, 1.0, 1.2, and 1.4, 1.6, 1.8, and 2.0, respectively. The market data are collected from SGX between 1 Aug 2019 and 30 Jun 2021, which covers the 2020 COVID-19 crash. The window size is six months. In the subfigures, the time window is indicated as *YYYYMMDD* of the starting date to *YYYYMMDD* of the ending date.

F. Spectral Sequences for Jan 2020 S&P 500 Crash

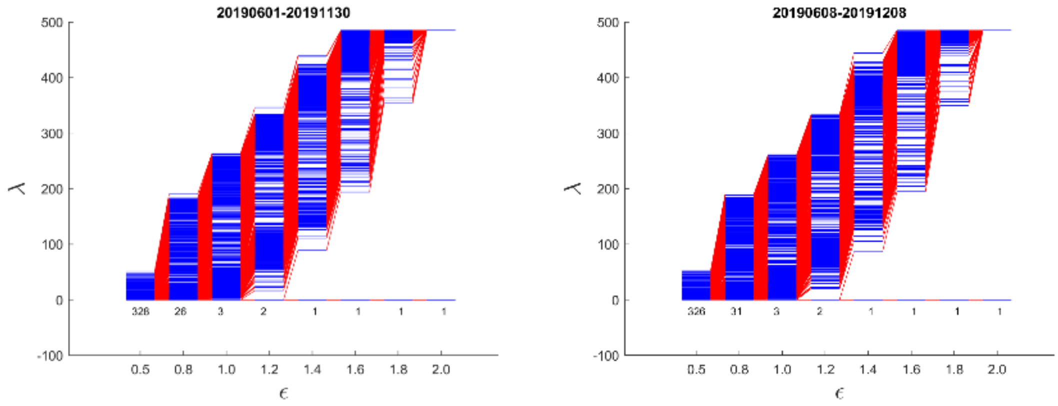

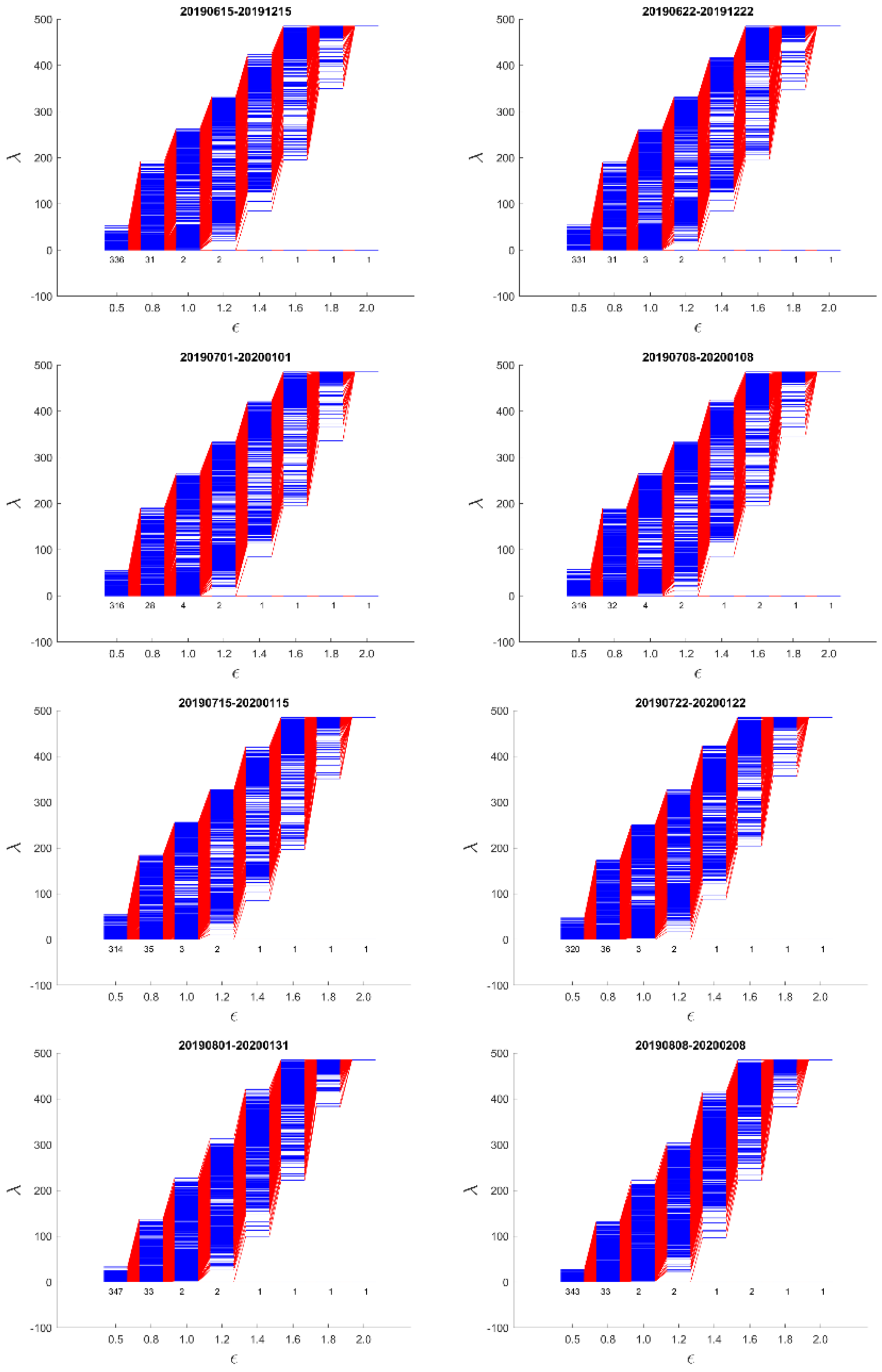
20190615-20191215
336 31 2 2 1 1 1 1
20190622-20191222
331 31 3 2 1 1 1 1
20190701-20200101
316 28 4 2 1 1 1 1
20190708-20200108
316 32 4 2 1 2 1 1
20190715-20200115
314 35 3 2 1 1 1 1
20190722-20200122
320 36 3 2 1 1 1 1
20190801-20200131
347 33 2 2 1 1 1 1
20190808-20200208
343 33 2 2 1 2 1 1
λ
ϵ
500
400
300
200
100
0
-100
0.5 0.8 1.0 1.2 1.4 1.6 1.8 2.0

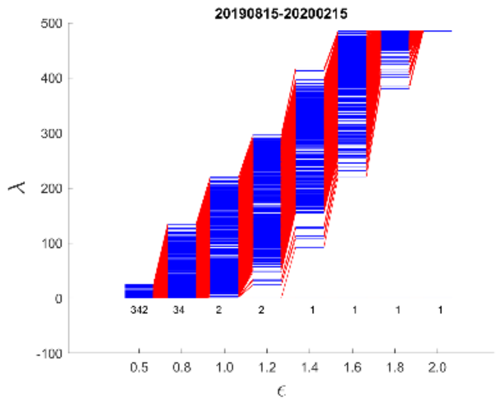
20190815-20200215
λ
ϵ

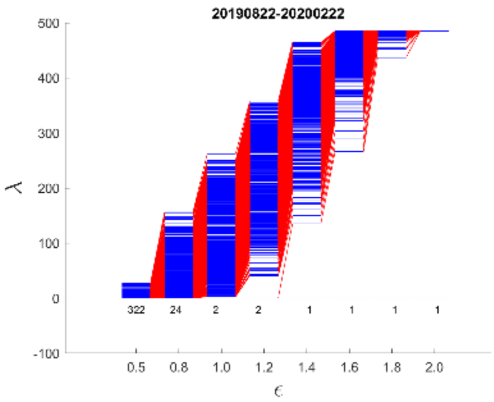
20190822-20200222
λ
ϵ

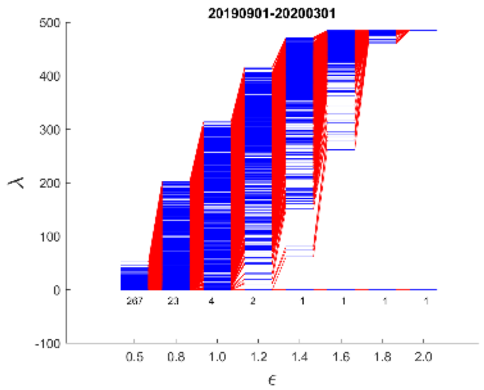
20190901-20200301
λ
ϵ

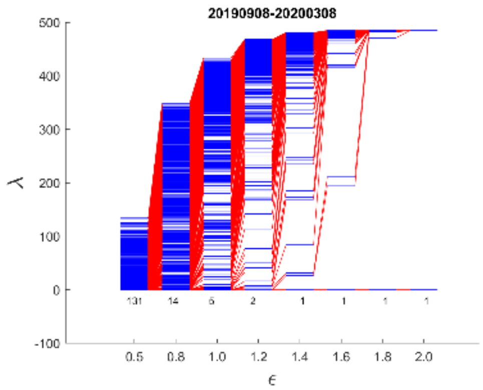
20190908-20200308
λ
ϵ

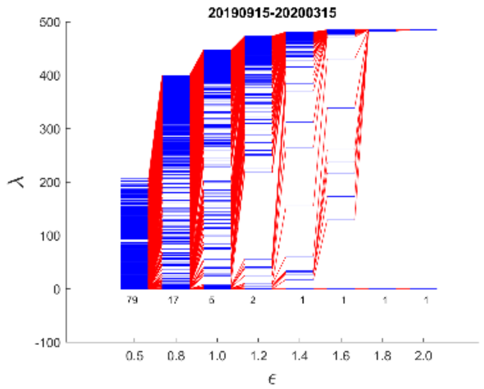
20190915-20200315
λ
ϵ

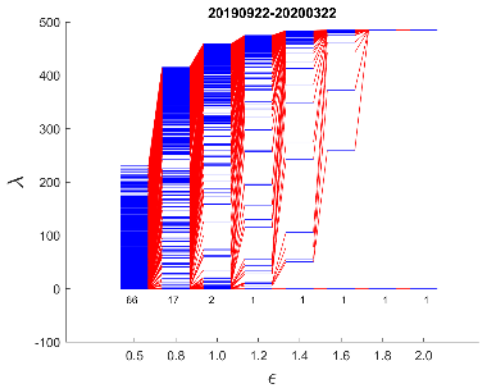
20190922-20200322
λ
ϵ

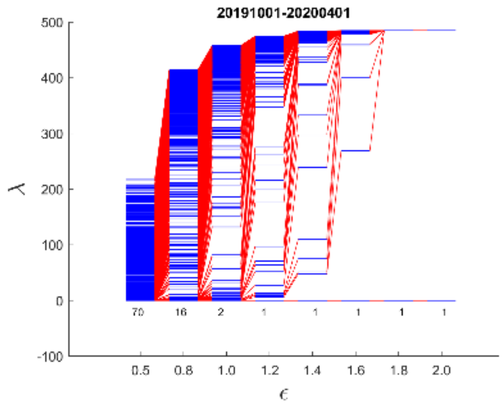
20191001-20200401
λ
ϵ

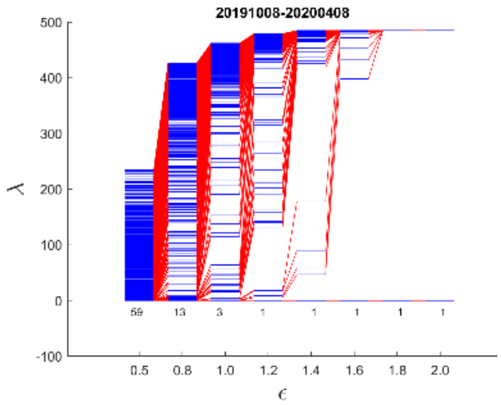
20191008-20200408
λ
ϵ

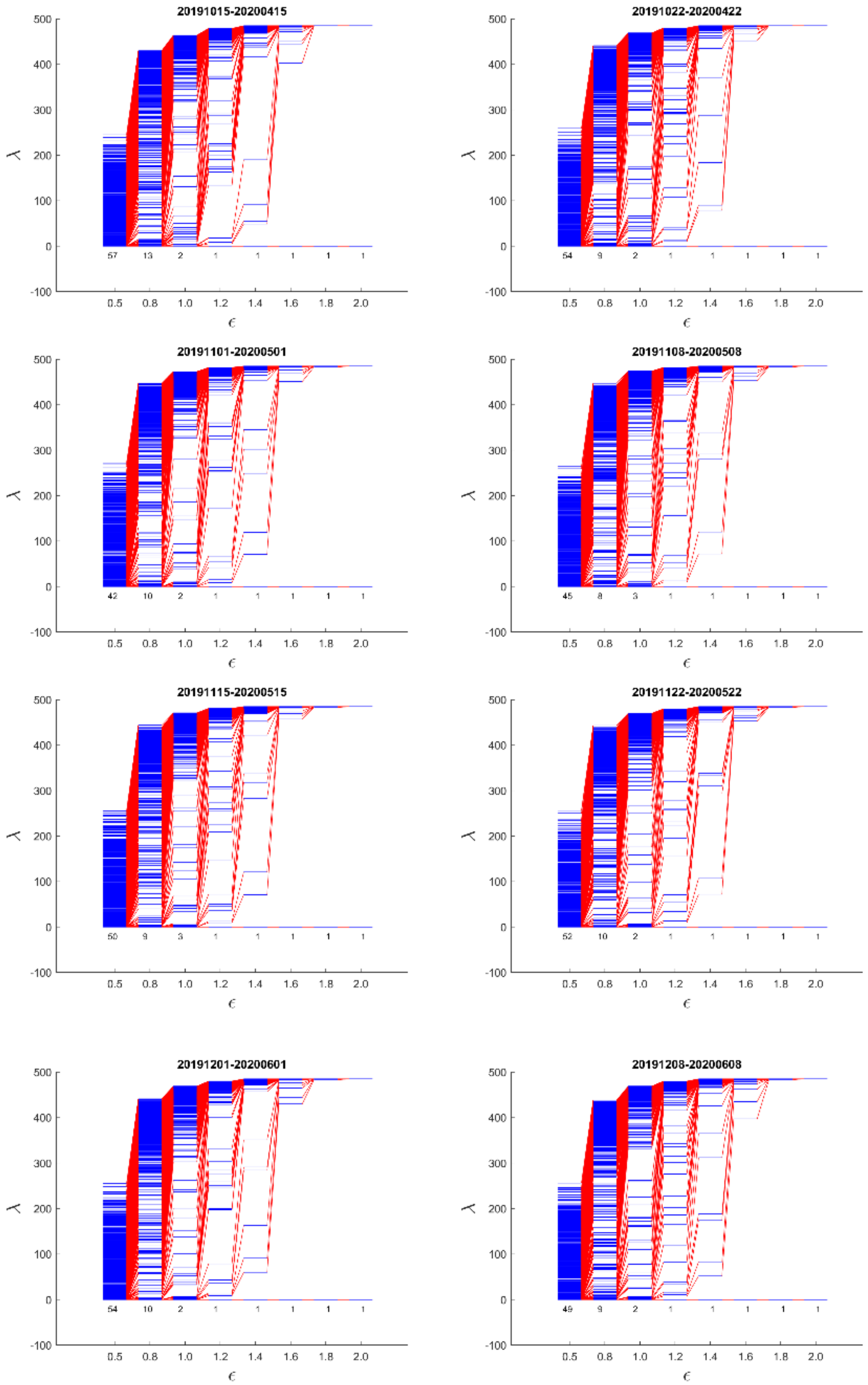
20191015-20200415
57 13 2 1 1 1 1 1
20191022-20200422
54 9 2 1 1 1 1 1
20191101-20200501
42 10 2 1 1 1 1 1
20191108-20200508
45 8 3 1 1 1 1 1
20191115-20200515
50 9 3 1 1 1 1 1
20191122-20200522
52 10 2 1 1 1 1 1
20191201-20200601
54 10 2 1 1 1 1 1
20191208-20200608
49 9 2 1 1 1 1 1
500 400 300 200 100 0 -100
λ
0.5 0.8 1.0 1.2 1.4 1.6 1.8 2.0
ϵ

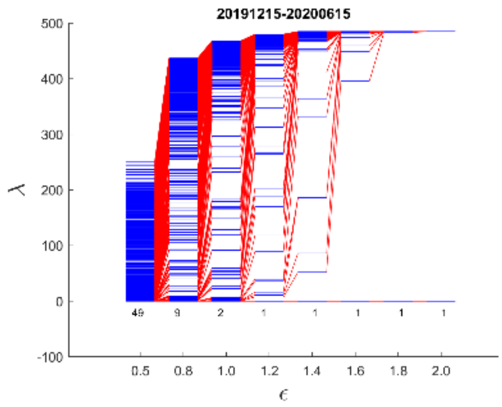
20191215-20200615
λ
ϵ
500 400 300 200 100 0 -100
0.5 0.8 1.0 1.2 1.4 1.6 1.8 2.0
49 9 2 1 1 1 1 1

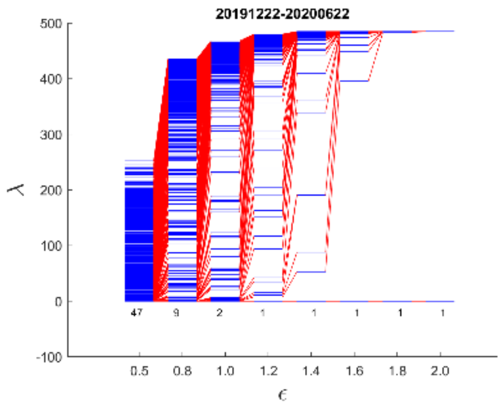
20191222-20200622
λ
ϵ
500 400 300 200 100 0 -100
0.5 0.8 1.0 1.2 1.4 1.6 1.8 2.0
47 9 2 1 1 1 1 1

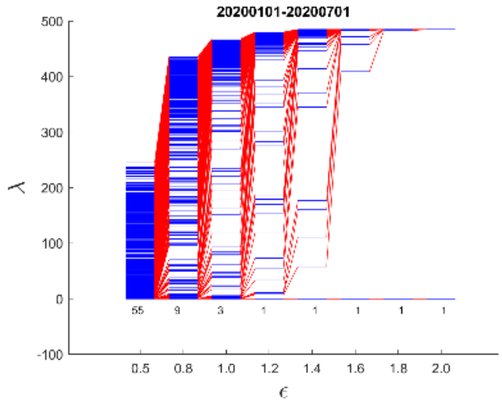
20200101-20200701
λ
ϵ
500 400 300 200 100 0 -100
0.5 0.8 1.0 1.2 1.4 1.6 1.8 2.0
55 9 3 1 1 1 1 1

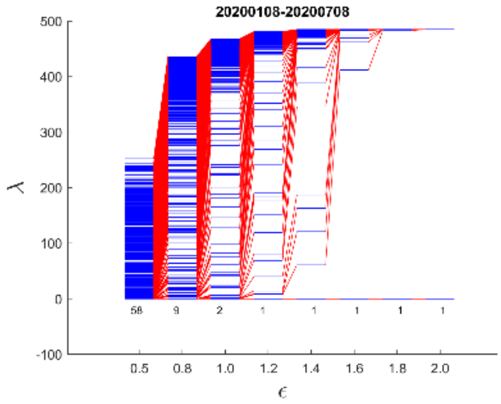
20200108-20200708
λ
ϵ
500 400 300 200 100 0 -100
0.5 0.8 1.0 1.2 1.4 1.6 1.8 2.0
58 9 2 1 1 1 1 1

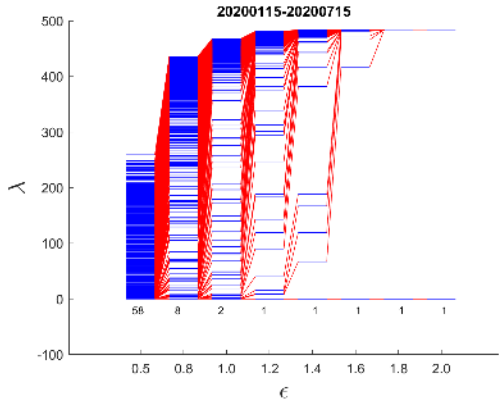
20200115-20200715
λ
ϵ
500 400 300 200 100 0 -100
0.5 0.8 1.0 1.2 1.4 1.6 1.8 2.0
58 8 2 1 1 1 1 1

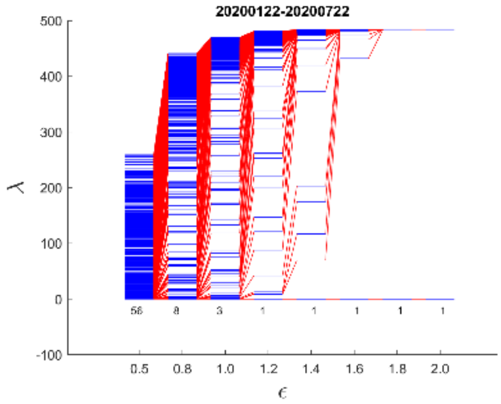
20200122-20200722
λ
ϵ
500 400 300 200 100 0 -100
0.5 0.8 1.0 1.2 1.4 1.6 1.8 2.0
56 8 3 1 1 1 1 1

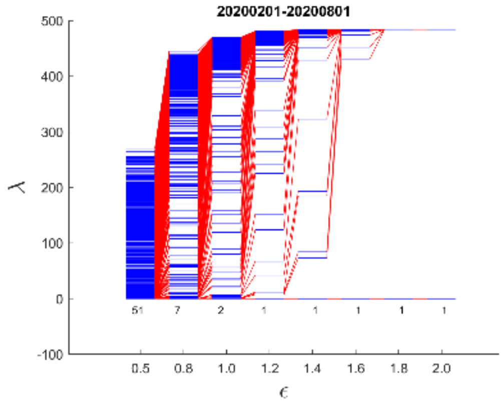
20200201-20200801
λ
ϵ
500 400 300 200 100 0 -100
0.5 0.8 1.0 1.2 1.4 1.6 1.8 2.0
51 7 2 1 1 1 1 1

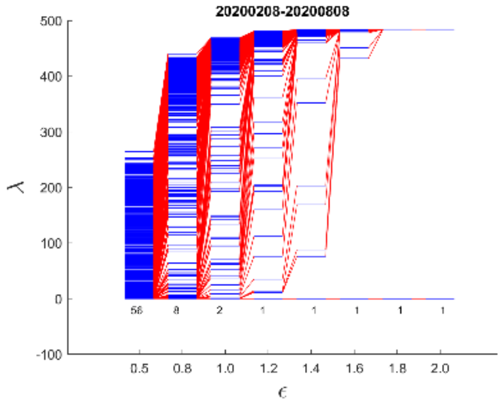
20200208-20200808
λ
ϵ
500 400 300 200 100 0 -100
0.5 0.8 1.0 1.2 1.4 1.6 1.8 2.0
56 8 2 1 1 1 1 1

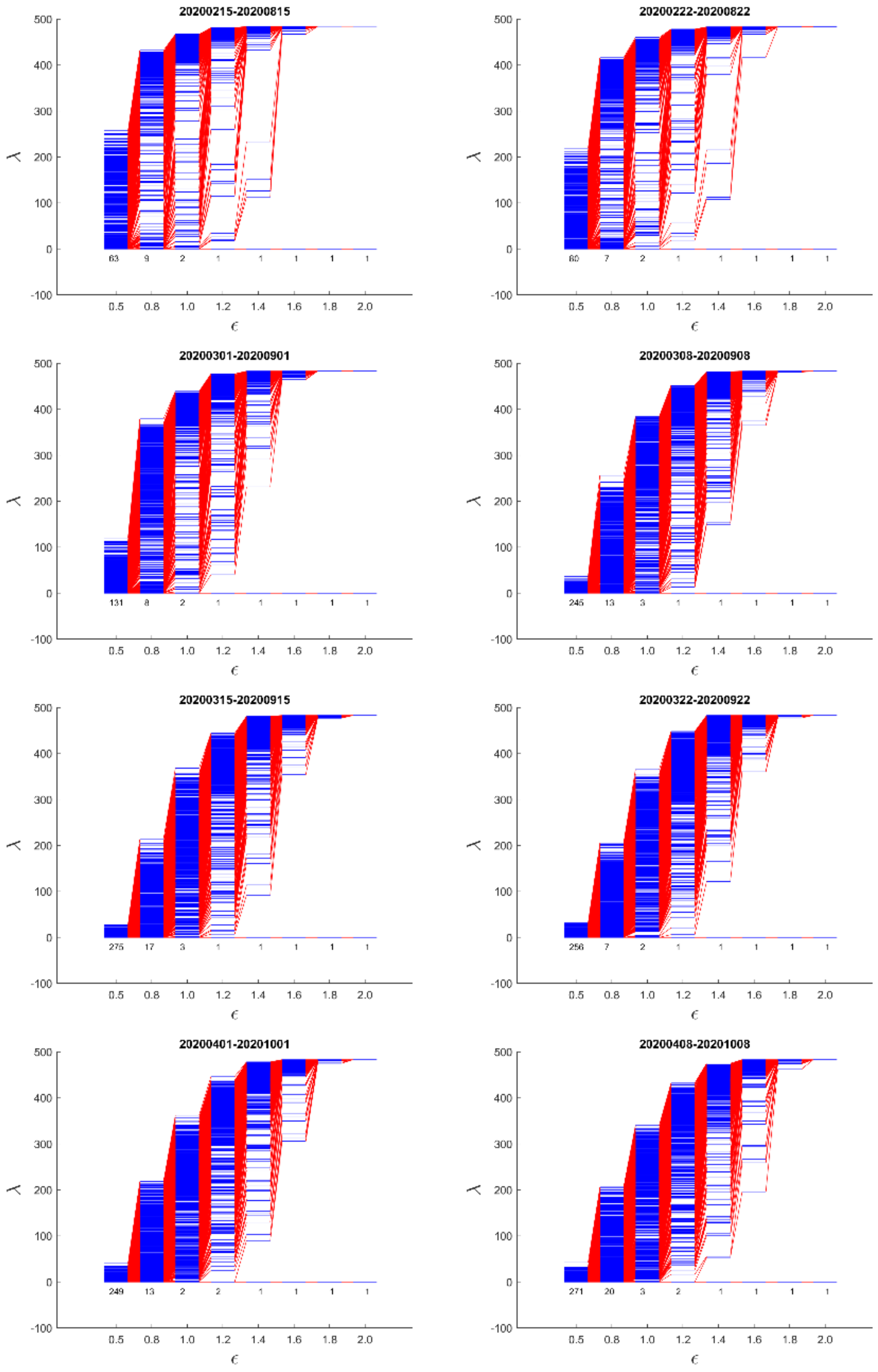
20200215-20200815
63 9 2 1 1 1 1 1
20200222-20200822
60 7 2 1 1 1 1 1
20200301-20200901
131 8 2 1 1 1 1 1
20200308-20200908
246 13 3 1 1 1 1 1
20200315-20200915
275 17 3 1 1 1 1 1
20200322-20200922
256 7 2 1 1 1 1 1
20200401-20201001
249 13 2 2 1 1 1 1
20200408-20201008
271 20 3 2 1 1 1 1
λ
500 400 300 200 100 0 -100
0.5 0.8 1.0 1.2 1.4 1.6 1.8 2.0
ϵ

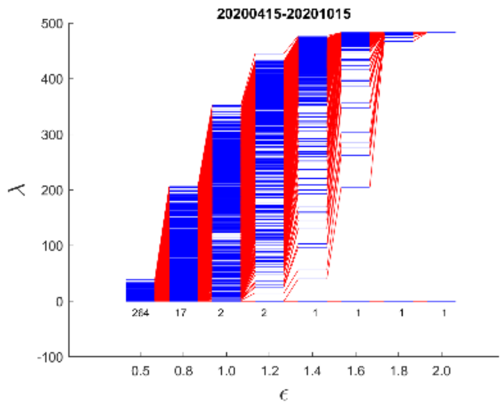
20200415-20201015

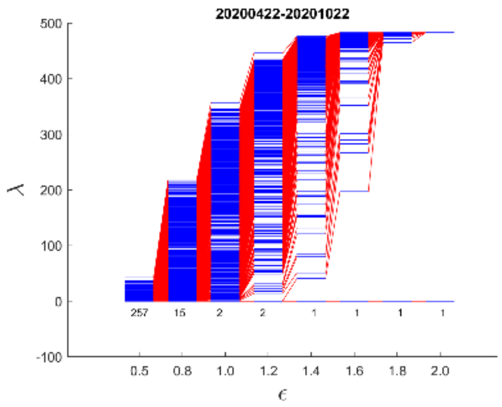
20200422-20201022

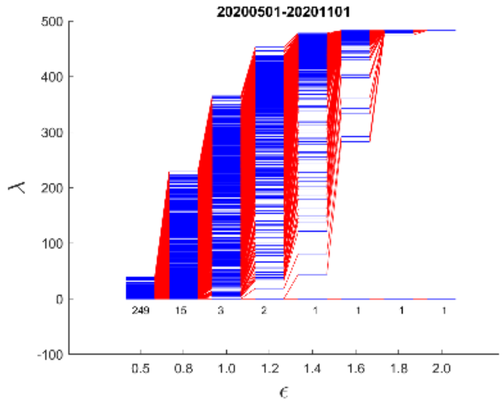
20200501-20201101

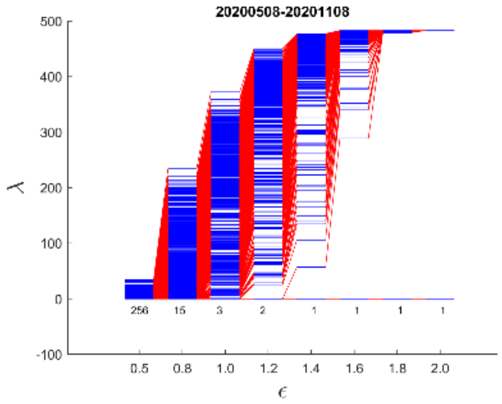
20200508-20201108

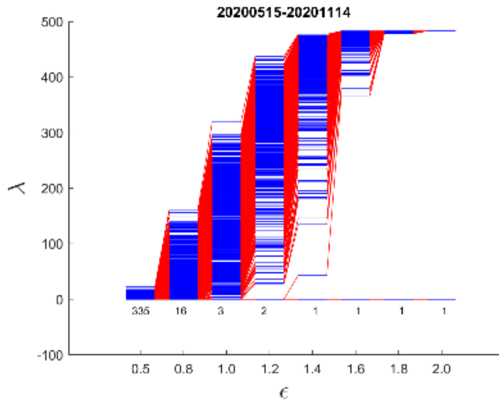
20200515-20201114

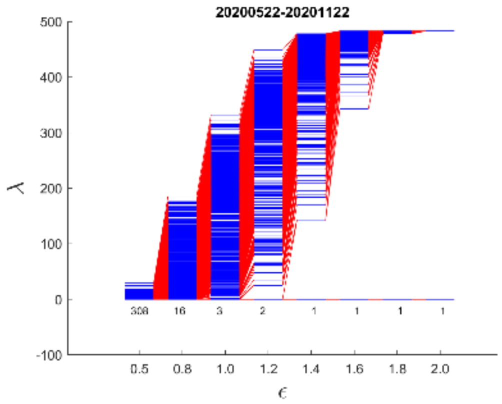
20200522-20201122

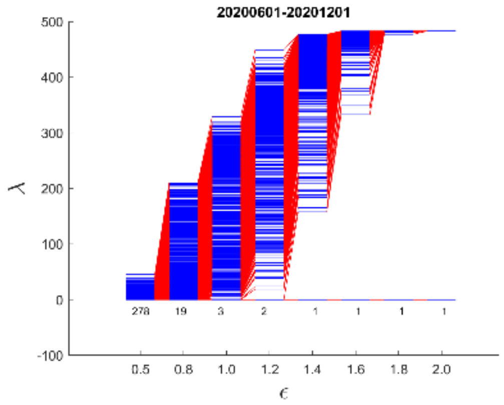
20200601-20201201

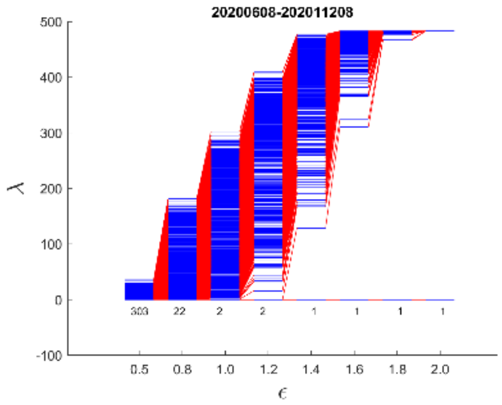
20200608-20201208

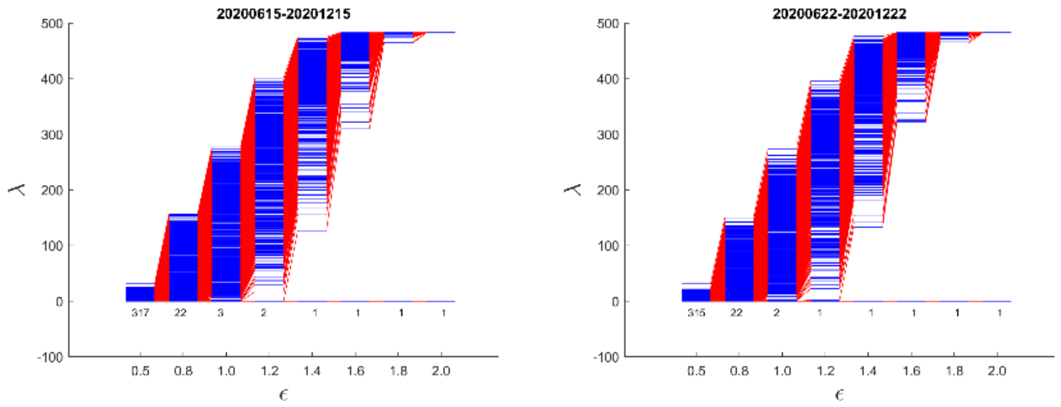

Figure F1: Index versus eigenvalues. We studied them at eight prescribed ϵ values, namely ϵ = 0.5, 0.8, 1.0, 1.2, and 1.4, 1.6, 1.8, and 2.0, respectively. The market data are collected from S&P 500 between 1 Jun 2019 and 30 Dec 2020, which covers the 2020 COVID-19 crash. The window size is six months. In the subfigures, the time window is indicated as *YYYYMMDD* of the starting date to *YYYYMMDD* of the ending date.

G. Neck and Bridging Components for the Mar 2020 TWSE COVID-19 Crash

Table G1: Components of the minor cluster in the four time windows at different filtration parameters. In this table, members of the minor cluster at a given ϵ are shown in italics, if they are found in the minor cluster of the succeeding ϵ, and shown in bold, if they are found in the minor cluster of the preceding ϵ. If a component is found across the minor clusters of the previous, present, and subsequent ϵ, it is shown in bold italics.

Time window	$\epsilon = 1.2$	$\epsilon = 1.4$	$\epsilon = 1.6$	$\epsilon = 1.8$
1 Aug 2019– 31 Jan 2020	-	1333.TWO, 2719.TWO, 2724.TWO, 3095.TWO, 3191.TWO, 3211.TWO, 3217.TWO, 3228.TWO, 3373.TWO, 3444.TWO, 4102.TWO, 4121.TWO, 4131.TWO, 4171.TWO, 4304.TWO, 4413.TWO, 4419.TWO, 4735.TWO, 4736.TWO, 5205.TWO, 5212.TWO, 5245.TWO, 5274.TWO, 5310.TWO, 5543.TWO, 6101.TWO, 6103.TWO, 6212.TWO, 6228.TWO, 6236.TWO, 6259.TWO, 6419.TWO, 6457.TWO, 6569.TWO, 8084.TWO, 8171.TWO, 8418.TWO	-	'1258.TWO', '1597.TWO', '2729.TWO', '3092.TWO', '3114.TWO', '3131.TWO', '3213.TWO', '3252.TWO', '3265.TWO', '3285.TWO', '3306.TWO', '3362.TWO', '3374.TWO', '3388.TWO', '3402.TWO', '3438.TWO', '3492.TWO', '3511.TWO', '3529.TWO', '3551.TWO', '3552.TWO', '3577.TWO', '3615.TWO', '3632.TWO', '3663.TWO', '3687.TWO', '3689.TWO', '4113.TWO', '4120.TWO', '4129.TWO', '4138.TWO', '4188.TWO', '4401.TWO', '4420.TWO', '4433.TWO', '4510.TWO', '4541.TWO', '4542.TWO', '4549.TWO', '4609.TWO', '4721.TWO', '4754.TWO', '4953.TWO', '4971.TWO', '5102.TWO', '5227.TWO', '5287.TWO', '5291.TWO', '5306.TWO', '5351.TWO', '5364.TWO', '5371.TWO', '5392.TWO', '5410.TWO', '5425.TWO', '5450.TWO', '5452.TWO', '5488.TWO', '5508.TWO', '5529.TWO', '6026.TWO', '6113.TWO', '6143.TWO', '6150.TWO', '6151.TWO', '6171.TWO', '6203.TWO', '6207.TWO', '6217.TWO', '6218.TWO',

				'6220.TWO', '6227.TWO', '6237.TWO', '6238.TWO', '6242.TWO', '6244.TWO', '6499.TWO', '6510.TWO', '8066.TWO', '8067.TWO', '8091.TWO', '8111.TWO', '8349.TWO', '8354.TWO', '8383.TWO', '8420.TWO', '8426.TWO', '8446.TWO', '8455.TWO'
22 Sep 2019–22 Mar 2020	*41*, *83*, *90*, *135*, *145*, *215*, *260*, *263*, *342*, *355*, *367*, *428*, *449*, *555*, *611*, *628*, 655	*3*, 40, **41**, *66*, **83**, 88, **90**, 97, ***135***, **145**, ***215***, 224, 231, 245, 255, **260**, **263**, 301, 302, **342**, 347, 351, **355**, **367**, **428**, 448, ***449***, 505, 514, 519, 530, 550, ***555***, 580, **611**, **628**, 653	**1333.TWO, 3095.TWO, *3373.TWO*, *4102.TWO*, 4413.TWO, *4419.TWO*, 6103.TWO, *6457.TWO***	**3373.TWO**, **4102.TWO**, **4419.TWO**, 5310.TWO, **6457.TWO**
15 Oct 2019–15 Apr 2020	3284.TWO, *4406.TWO*, 5398.TWO, 8415.TWO	2724.TWO, 3095.TWO, 4102.TWO, ***4406.TWO***, *4413.TWO*, *4419.TWO*, 4429.TWO, 5205.TWO, 5310.TWO, 5543.TWO, *6103.TWO*, *8077.TWO*	**4406.TWO**, ***4413.TWO***, ***4419.TWO***, ***6103.TWO***, ***8077.TWO***	3522.TWO, **4413.TWO**, **4419.TWO**, 5314.TWO, **6103.TWO**, **8077.TWO**
1 Apr 2020–30 Sep 2020	-	1584.TWO, 1586.TWO, 2067.TWO, 2718.TWO, 2724.TWO, 2924.TWO, 3067.TWO, 3073.TWO, 3078.TWO, 3085.TWO, 3093.TWO, 3095.TWO, 3105.TWO, 3115.TWO, 3162.TWO, 3188.TWO, 3218.TWO, 3228.TWO, 3268.TWO, 3272.TWO, 3285.TWO, 3287.TWO, 3293.TWO, 3310.TWO, 3325.TWO, 3339.TWO, 3379.TWO, 3465.TWO, 3484.TWO, 3490.TWO, 3498.TWO, 3516.TWO, 3540.TWO, 3581.TWO, 3594.TWO, 3629.TWO, 3666.TWO, 3691.TWO, 3707.TWO, 4198.TWO, 4303.TWO, 4413.TWO, 4429.TWO, 4523.TWO, 4530.TWO, 4543.TWO, 4549.TWO, 4609.TWO, 4716.TWO, 4747.TWO, 4924.TWO, 4933.TWO, 4950.TWO, 5205.TWO, 5206.TWO, 5310.TWO, 5321.TWO, 5324.TWO, 5355.TWO, 5432.TWO, 5452.TWO, 5457.TWO, 5460.TWO, 5468.TWO, 5474.TWO, 5475.TWO, 5508.TWO, 5529.TWO, 5543.TWO, 5609.TWO, 5820.TWO, 5902.TWO, 6103.TWO, 6125.TWO, 6126.TWO, 6134.TWO, 6140.TWO, 6148.TWO, 6187.TWO, 6194.TWO,	-	3064.TWO, 3085.TWO, 3095.TWO, 3230.TWO, 3465.TWO, 3632.TWO, 3707.TWO, 4406.TWO, 4530.TWO, 4804.TWO, 5475.TWO, 5601.TWO, 6101.TWO, 6113.TWO, 6148.TWO, 6233.TWO, 6246.TWO, 6264.TWO, 6462.TWO

		6195.TWO, 6198.TWO,<br>6199.TWO, 6204.TWO,<br>6212.TWO, 6220.TWO,<br>6222.TWO, 6228.TWO,<br>6229.TWO, 6233.TWO,<br>6236.TWO, 6244.TWO,<br>6246.TWO, 6247.TWO,<br>6259.TWO, 6264.TWO,<br>6404.TWO, 6417.TWO,<br>6465.TWO, 6469.TWO,<br>6510.TWO, 6538.TWO,<br>8047.TWO, 8077.TWO,<br>8080.TWO, 8083.TWO,<br>8085.TWO, 8121.TWO,<br>8171.TWO, 8415.TWO,<br>8432.TWO, 8435.TWO		
